# The prevalence and risk factors of functional dyspepsia among adults in low- and middle-income countries: An international cross-sectional study

**DOI:** 10.1097/MD.0000000000035437

**Published:** 2023-10-06

**Authors:** Ahmad Yamen Arnaout, Tala Jouma Alhejazi, Yaman Nerabani, Ola Hamdan, Khaled Arnaout, Ibrahim Arnaout, Ziad aljarad

**Affiliations:** a Faculty of Medicine, University of Aleppo, Aleppo, Syrian Arab Republic; b MD, MSc, PhD Gastroenterologist, University of Aleppo, Aleppo, Syrian Arab Republic.

**Keywords:** international cross-sectional study, functional dyspepsia, prevalence, risk factors

## Abstract

Dyspepsia is one of the most common chronic digestive diseases, which is due to underlying organic causes that can be detected, or causes that cannot be detected called functional dyspepsia (FD). There is no epidemiological study to date that measures the prevalence and risk factors of the FD in low- and middle-income countries, so this international cross-sectional study was conducted in 15 low- and middle-income countries from data previously published its protocol NCT05340400. Participants were recruited in the period from 22/April/2022 to 14/June/2022. The diagnosis of the FD was according to clinical manifestations. We determined the physical activity, daily stress, and fatigue of the participants. A large number of collaborators were chosen from different regions and institutions within each country to achieve diversity within the sample and reduce the probability of bias. Our study concluded that the prevalence of FD is much higher in low- and middle-income countries 37.9% [6.2%–44.2%], compared to high-income countries 10% [8%–12%], with a higher rate among the Afro-Caribbean race 47.9%. Sudan obtained the highest prevalence 44.3%, then Egypt 41.4%, while the lowest prevalence was in Algeria 25.7%. Moreover, there are many risk factors, including modifiable ones, such as severe stress, chronic fatigue, smoking, abnormal BMI, insufficient or too many hours of sleep, and previous infection with Covid-19, and non-modifiable ones such as advanced age, chronic diseases, and female sex. Highlighting the prevalence and increasing risk factors of FD in low- and middle-income countries should draw the attention of those responsible for health care in these countries and reduce the risk factors.

## 1. Introduction

Indigestion is one of the most common chronic digestive diseases, which is due to underlying organic causes that can be detected, or causes that cannot be detected called functional dyspepsia (FD).

In a study conducted in the United States, the United Kingdom and Canada, an average prevalence rate of 10% was reported, with a higher prevalence in the USA, approximately 12%.^[1]^

A meta-analysis of epidemiological studies revealed that female sex, smoking, use of non-steroidal anti-inflammatory drugs (NSAIDs), and H. pylori infection were risk factors for FD, but with a modest association between them.^[2]^

However, these epidemiological studies to calculate prevalence and risk factors have not yet included low- and middle-income countries such as Egypt, Syria, Sudan, Algeria, and many others. Moreover, the expected increase in the prevalence of FD in the world in general and in the low- and middle-income countries in particular, our study was established aiming to assess and detect latent potential and apparent risk factors.

## 2. Methods

### 2.1. Study design

This cross-sectional study was conducted from PRIBS Study data between April 2022 and June 2022. The study included 5506 participants from 15 countries. The study was preregistered on the NCT05340400. and followed the Strengthening in Reporting Observational studies in Epidemiology (STROBE) guidelines for cross-sectional study.^[3]^

### 2.2. Setting

The study protocol was according to the ethical guidelines of the 1964 Declaration of Helsinki and its later update. Additionally, The Ethical Review Board of Faculty of Medicine, University of Aleppo, Syria has also approved the study. Moreover, ethical approval was provided by national leaders in each enrolled country. Then, the data was collected using face-to-face interview to give an explanation for the questionnaire.

### 2.3. Patient and public involvement

The inclusion criteria were: adults willing to participate in this survey who were 18 years of age or older. Exclusion criteria were: any participant who was diagnosed with poorly-controlled hyperthyroidism, poorly-controlled hypothyroidism, poorly-controlled hyperparathyroidism, paralysis, or parasitic diseases. Moreover, any presence (or suspicion) of liver disease, celiac disease, inflammatory bowel disease (Crohn disease or Ulcerative colitis), lactose intolerance, or cancer or tumor in the digestive tract in their clinical history. No formal sample size was calculated for this study.

The total number of participants in the study was 5506, from 15 low- and middle-income countries. The number of participants from Syria was 2909, Egypt 536, Sudan 536, Pakistan 380, Libya 222, Algeria 222, Jordan 176, Iraq 125, India 102, Yemen 72, Palestine 69, and the rest from Morocco, Serbia, Bangladesh, and Saudi Arabia. With the participation of 148 data collectors from different cities and institutions from the participating countries. The participants volunteered to participate by agreeing to the investigation and filling out their medical data.

### 2.4. Variables and measurement

The questionnaire was divided into separate sections, the first one was covering the socio-demographic characteristics such as (age, gender, body mass index [BMI]). We used the American Society of Anaesthesiologists Classification to categorize the people into 5 grades according to their health situation.^[4]^ Participants were also asked about their co-morbidities.

While the diagnosis of the epigastric pain syndrome was by identifying the presence of the epigastric pain, epigastric burning, or both at least once a week in the last 3 months. Furthermore, we diagnosed the postprandial distress syndrome in the event of suffering from postprandial fullness, early satiety, or both in at least 3 days per week in the last 3 months. The diagnosis of FD was by identifying either epigastric pain syndrome, postprandial distress syndrome, or both.

The physical activity of the participants was determined according to Global Physical Activity Score of the World Health Organization (WHO). This score consists of 3 parts: the quantity of effort spent at daily work (vigorous or moderate-intensity activity), and traveling from one area to another, and the third part the type of sport in daily routine. Then, physical activity classified into required or not.^[5]^

Daily stress was evaluated with the perceived stress scale (PSS), the most widely used measure of global perceived stress, and is a robust predictor of health and disease. The total score is calculated on the basis of the answers to a series of questions based on monthly stress, and the participant health status. PSS is a summary measure of ten items (range 0–4 points for every item). It is classified into low (PSS 0–13), moderate (PSS 14–26), and high perceived stress (PSS 27–40).^[6]^

Fatigue was measured using the Chalder Fatigue Scale (CFQ), a questionnaire for measuring the extent and severity of fatigue within both clinical and non-clinical, epidemiological studies.^[7]^

Diet and daily habits, including smoking and alcohol consuming, were evaluated. Participants were classified into 4 sections according to WHO Smoking and Tobacco Use Policy. A daily smoker is someone who smokes any tobacco product at least once a day, and an occasional smoker is someone who smokes, but not every day.^[8]^

Each question was explained to the participant separately by the collaborator.

### 2.5. Bias

A large number of collaborators were chosen from different regions and institutions within each country to achieve diversity within the sample and reduce the probability of bias. We trained the collaborators with a course explaining each section of the questionnaire and how to present it to the participants. The tutorial videos were uploaded in Arabic and English on YouTube. We also translated the questionnaire into Arabic by 2 Arab doctors separately and simultaneously and it was checked by another, because most of the participating countries are from Arab countries.

### 2.6. Statistical methods

Data were analyzed using the SPSS PC version 24.0 statistical software. Descriptive statistics (mean, standard deviation, frequencies, and percentages) were used to describe the quantitative and categorical variables. Moreover, the Chi-square test was used for each variable only if the sample number exceeded 100 to observe the association between the categorical study and outcome variables. The quantitative variables such as age, and BMI were divided into categories and then we equated the chi-test Square. We calculated the prevalence for each country if the country participants exceed 200. All the results of the statistical inference tests were interpreted to a 95% confidence level, that is, the significance level of 0.05 was used with 2-tailed hypothesis. The normal distribution suitability of the numerical variables was tested with the Shapiro–Wilk test.

## 3. Results

### 3.1. Participants and descriptive data

The prominent ethnicity was Caucasian (85.3%), then Asian (9%), Afro-Caribbean (4.9%), and other ethnicities (0.8%). The mean age of the participants was 32.7, with a standard deviation of 14.3. Most of participants were female 58.2% (41.8% male). And there were no missed data.

### 3.2. Prevalence of FD

The average prevalence of FD was 37.9% [6.2%–44.2%], with a higher rate among the Afro-Caribbean race 47.9%. Sudan obtained the highest prevalence 44.3%, then Egypt 41.4%, while the lowest prevalence was in Algeria 25.7% Table 1.

**Table 1:** Prevalence of functional dyspepsia according to the population based of the participants.

Category	Sub- category	Participants without FD	Participants with FD	Total
Ethnicity				
	Caucasian (n, %)	2897 (61.7%)	1796 (38.3%)	4695
	Afro-Caribbean (n, %)	139 (52.1%)	128 (47.9%)	267
	Asian (n, %)	358 (71.9%)	140 (28.1%)	498
	Hispanic (n, %)	2 (100%)	Not Counted	2
	Other (n, %)	24 (55.8%)	Not Counted	43
	Total	3420 (62.1%)	2085 (37.9%)	5506
Country				
	Syria (n, %)	1818 (62.5%)	1091 (37.5%)	2909
	Egypt (n, %)	314 (58.6%)	222 (41.4%)	536
	Pakistan (n, %)	266 (70%)	114 (30%)	380
	Sudan (n, %)	299 (55.7%)	238 (44.3%)	537
	Algeria (n, %)	165 (74.3%)	57 (25.7%)	222
	Total*	3420 (74.8%)	2085 (37.9%)	5505

FD = functional dyspepsia.

* For all study participants, even from countries with <200 participants.

### 3.3. Risk Factors of FD

#### 3.3.1. Sociodemographic risk factors.

The prevalence of FD increased significantly with age, reaching 48.3% at age more than 60 years. Females were more likely to have the disease than males (41.9% vs 32.2%, *P* value < .001). Moreover, FD prevalence increased among abnormal participants’ BMI (*P* value < .001). Healthy people were less likely to have the disease than people with mild and sever systemic diseases (33.3%, 46.8%, 53.8%, respectively *P* value < .001). Finally, the prevalence of FD was higher among rural participants in comparison with urban participants (41.4% vs 37.4%, *P* value = .001) Table 2.

**Table 2:** Correlation of FD according to the sociodemographic of the participants.

Category	Sub- category	Participants without FD	Participants with FD	Total	Pearson Chi-Square	*P* value
Age					82.869	.000
	18–30 (n, %)	2259 (66.6%)	1132 (33.4%)	3391		
	31–40 (n, %)	412 (58.8%)	289 (41.2%)	701		
	41–50 (n, %)	325 (54%)	277 (46%)	602		
	51–60 (n, %)	253 (52.7%)	227 (47.3%)	480		
	More than 60 (n, %)	171 (51.7%)	160 (48.3%)	330		
Gender					54.041	.000
	Male (n, %)	1560 (67.8%)	741 (32.2%)	2301		
	Female (n, %)	1860 (58.1%)	1344 (41.9%)	3204		
BMI according WHO classification					39.684	.000
	Underweight < 18.5 (n, %)	223 (59.8%)	150 (40.2%)	373		
	Normal Range [18.5–24.9] (n, %)	1761 (65.1%)	943 (34.9%)	2704		
	Overweight [25.0–29.9] (n, %)	940 (61.4%)	591 (38.6%)	1531		
	Obese Class I [30–34.9] (n, %)	318 (53.7%)	274 (46.3%)	592		
	Obese Class II [35–39.9] (n, %)	94 (56%)	74 (44%)	168		
	Obese Class III > 40 (n, %)	21 (42.9%)	28 (57.1%)	49		
ASA grade					117.737^a^	.000
	ASA I (n, %)	2549 (66.7%)	1275 (33.3%)	3824		
	ASA II (n, %)	745 (53.2%)	656 (46.8%)	1401		
	ASA III (n, %)	114 (46.2%)	133 (53.8%)	247		
	ASA IV (n, %)	9 (33.3%)	18 (66.7%)	27		
	ASA V (n, %)	Not Calculated	Not Calculated	6		
Geographic					13.584	.001
	Urban life (n, %)	2779 (62.6%)	1660 (37.4%)	4439		
	Rural life (n, %)	568 (58.6%)	402 (41.4%)	970		
	Nomad life (n, %)	Not Calculated	Not Calculated	96		

ASA I: Healthy person. ASA II: Mild systemic disease. ASA III: Severe systemic disease. ASA IV: Severe systemic disease that is a constant threat to life. ASA IV: A moribund person who is not expected to survive without the operation. ASA V: A declared brain-dead person whose organs are being removed for donor purposes.

ASA = American Society of Anesthesiologists Classification, BMI = body mass index, IBS = irritable bowel syndrome.

#### 3.3.2. Co-morbidities risk factors.

The prevalence of FD was significantly higher in participants with certain co-morbidities (Hypertension requiring medication, diabetes mellitus, autoimmune diseases, headache or migraine, anemia, allergies to certain substances, asthma, ischemic heart disease, and endometriosis). Regarding the diagnosis of COVID-19 within the last 12 months, people were more likely to have FD if they have been tested positive than negative (41.1% vs 35.3% *P* value < .001). Also, the prevalence of the disease increased with the presence of previous abdominal surgery. There is a significant increase in prevalence with fatigue severity (low = 24.6%, moderate = 40.3%, and severe = 59.2%, *P* value < .001). Also, there is a significant increase in prevalence in the participants with high stress compared to moderate and low stress (51.7%, 37.0%, 21.3%, respectively, *P* value < .001) Table 3.

**Table 3:** Correlation of FD according to comorbidities.

Category	Sub- category	Participants without FD	Participants with FD	Total	Pearson Chi-Square	*P* value
GERD (n, %)	Yes	570 (39.3%)	881 (60.7%)	1451	436.913	.000
	No	2850 (70.3%)	1204 (29.7%)	4054		
Hypertension requiring medication (n, %)	Yes	294 (48.4%)	313 (51.6%)	607	54.343	.000
	No	3126 (63.8%)	1772 (36.2%)	4898		
Diabetes Mellitus (n, %)	Yes	194 (48.4%)	207 (51.6%)	401	34.733	.000
	No	3226 (63.2%)	1878 (36.8%)	5104		
Autoimmune diseases (n, %)	Yes	73 (51%)	70 (49%)	143	7.655	.006
	No	3347 (62.4%)	2015 (37.6%)	5362		
Headache or migraine (n, %)	Yes	268 (47.6%)	295 (52.4%)	563	56.217	.000
	No	3152 (63.8%)	1790 (36.2%)	4942		
Chronic immunosuppression* (n, %)	Yes	10 (43.5%)	13 (56.5%)	23*	3.413	.065
	No	3410 (62.2%)	2072 (37.8%)	5482		
Anemia (n, %)	Yes	358 (49.9%)	359 (50.1%)	717	52.104	.000
	No	3062 (64%)	1726 (36%)	4788		
Allergic to certain substances (n, %)	Yes	309 (50.9%)	298 (49.1%)	607	36.495	.000
	No	3111 (63.5%)	1787 (36.5%)	4898		
COPD (n, %)*	Yes	25 (50%)	25 (50%)	50	3.153	.076
	No	3395 (62.2%)	2060 (37.8%)	5455		
Asthma (n, %)	Yes	142 (53.4%)	124 (46.6%)	266	9.078	.003
	No	3278 (62.6%)	1961 (37.4%)	5239		
Ischemic heart disease (n, %)	Yes	53 (49.1%)	55 (50.9%)	108	7.975	.005
	No	3367 (62.4%)	2030 (37.6%)	5397		
Urinary problems (n, %)	Yes	111 (49.1)	115 (50.9%)	226	16.954	.000
	No	3309 (62.7%)	1970 (37.3%)	5279		
Endometriosis (n, %)	Yes	13 (41.9%)	18 (58.1%)	31	5.401	.020
	No	3407 (62.2%)	2067 (37.8%)	5474		
Past history of COVID-19 infection (within the last 12 mo) (n, %)	Yes	1433 (58.9%)	1002 (41.1%)	2435	19.906	.000
	No	1987 (64.7%)	1083 (35.3%)	3070		
Abdominal surgery/laparotomy (n, %)	Yes	584 (52%)	539 (48%)	1123	61.427	.000
	No	2836 (64.7%)	1546 (35.3%)	4382		
The Chalder fatigue scale						
	Low Fatigue (n, %)	1122 (75.4%)	366 (24.6%)	1488	223.860	.000
	Moderate Fatigue (n, %)	2079 (59.7%)	1401 (40.3%)	3480		
	Severe Fatigue (n, %)	219 (40.8%)	318 (59.2%)	537		
Perceived stress scale						
	Low stress (n, %)	369 (78.7%)	10 (21.3%)	469	122.586	.000
	Moderate stress (n, %)	2655 (63.0%)	1561 (37.0%)	4216		
	High stress (n, %)	396 (48.3%)	424 (51.7%)	820		

COPD = chronic obstructive pulmonary disease, EPS = epigastric pain syndrome, GERD = Gastro-esophagus retardation disease, IBS = irritable bowel syndrome, PPDS = postprandial distress syndrome.

#### 3.3.3. Habits and field of work risk factors.

The prevalence of FD significantly increased among participants who work in the fields of Informatics, technology, computer engineer, teaching, and home economics. On the other hand, it decreased among physicians and participants who do not work (*P* value < .05) Table 4.

**Table 4:** Correlation of FD according to the field of work or profession of the participants.

Category	Sub- category	Participants without FD	Participants with FD	Total	Pearson Chi-Square	*P* value
Agricultural projects and natural resources (e.g., farmer) (n, %)	Yes	66 (58.4%)	47 (41.6%)	113	.678	.410
	No	3354 (62.2%)	2038 (37.8%)	5392		
Civil engineer (n, %)	Yes	93 (61.6%)	58 (38.4%)	151	.019	.891
	No	3327 (62.1%)	2027 (37.9%)	5354		
Informatics, technology or computer engineer..etc (n, %)	Yes	114 (60%)	76 (40%)	190	.008	.014
	No	3306 (62.2%)	2009 (37.8%)	5315		
Commercial companies and offices (n, %)	Yes	119 (61%)	76 (39%)	195	.104	.747
	No	3301 (62.2%)	2009 (37.8%)	5310		
Physicians (n, %)	Yes	408 (67%)	201 (33%)	609	6.901	.009
	No	3012 (61.5%)	1884 (38.5%)	4896		
Nurses (n, %)	Yes	102 (56.4%)	79 (43.6%)	181	2.650	.104
	No	3318 (62.3%)	2006 (37.7%)	5324		
Home Economics (n, %)	Yes	117 (49.6%)	119 (50.4%)	236	16.502	.000
	No	3303 (62.7%)	1966 (37.3%)	5269		
Industry (n, %)	Yes	67 (58.8%)	47 (41.2%)	114	.556	.456
	No	3353 (62.2%)	2038 (37.8%)	5391		
Teaching (schools, Universities.) (n, %)	Yes	254 (55.8%)	201 (44.2%)	455	8.370	.004
	No	3166 (62.7%)	1884 (37.3%)	5050		
Working (n, %)	Yes	1935 (60.2%)	1277 (39.8%)	3212	11.614	.001
	No	1485 (64.8%)	808 (35.2%)	2293		

FD = functional dyspepsia.

By talking about smoking status and daily diet, smokers were more likely to have the disease regardless how much they smoke and even if they quit smoking. Drinking coffee and its derivatives also increased the prevalence of the disease (40.2% vs 35.9%, *P* value = .001). While Carbonated drinks decreased it (34% vs 38.4%, *P* value = .045).

Participants with intermediate sleeping hours (6–8 hours) got the lowest prevalence of FD, however, more than 8 hours or <6 increased the disease (35.3%, 41.5, 42.5 respectively, *P* value < .001) Table 5.

**Table 5:** Correlation of FD according to habits.

Category	Sub- category	Participants without FD	Participants with FD	Total	Pearson Chi-Square	*P* value
Global Physical Activity Score						
	Physical activity is not required (Score > 600) (n, %)	578 (60%)	386 (40%)	964	3.417	.181
	Physical activity is required (Score < 600) (n, %)	2840 (62.6%)	1696 (37.4%)	4536		
Participant Predominant Food Pattern						
	Fatty food (n, %)	320 (58.3%)	229 (41.7%)	549	6.747	.240
	Carbohydrates (n, %)	684 (62.1%)	418 (37.9%)	1102		
	Protein food (n, %)	227 (59.9%)	152 (40.1%)	379		
	High-fiber foods (n, %)	197 (60.8%)	127 (39.2%)	324		
	Other (n, %)	39 (68.4%)	18 (31.6%)	57		
	Diverse (n, %)	1953 (63.1%)	1141 (36.9%)	3094		
Smoking						
	A daily smoker (n, %)	504 (54.2%)	426 (45.8%)	930	33.913	.000
	An occasional smoker (n, %)	384 (61.2%)	243 (38.8%)	627		
	Ex-smoker (n, %)	172 (62.5%)	103 (37.5%)	275		
	nonsmoker (n, %)	2360 (64.3%)	1312 (35.7%)	3672		
Drinking alcohol						
	Not Drinking (n, %)	3276 (62.5%)	1969 (37.5%)	5245	7.075	.132
	In moderation (n, %)	136 (56%)	107 (44%)	243		
Sleeping hours						
	<6 (n, %)	605 (57.5%)	447 (42.5%)	1052	26.970	.000
	Between 6–8 (n, %)	2180 (64.7%)	1187 (35.3%)	3367		
	More than 8 (n, %)	635 (58.5%)	450 (41.5%)	1085		
kind of stimulants that participants usually drink?						
Tea (n, %)	Yes	1705 (60.9%)	1094 (39.1%)	2799	3.069	.080
	No	1649 (63.2%)	959 (36.8%)	2608		
Coffee and its derivatives (n, %)	Yes	1572 (59.8%)	1055 (40.2%)	2627	10.410	.001
	No	1782 (64.1%)	998 (35.9%)	2780		
Mate (drink) (n, %)	Yes	313 (60.7%)	203 (39.3%)	516	.456	.500
	No	3041 (62.2%)	1850 (37.8%)	4891		
Energy Drinks (n, %)	Yes	169 (61.7%)	105 (38.3%)	274	.015	.902
	No	3185 (62%)	1948 (38%)	5133		
Carbonated drinks (n, %)	Yes	350 (66%)	180 (34%)	530	4.006	.045
	No	3004 (61.6%)	1873 (38.4%)	4877		

FD = functional dyspepsia.

## 4. Discussion

### 4.1. Prevalence of FD

Our study included 5506 participants from 15 low- and middle-income countries. The mean prevalence of FD in our sample was 37.9%, with higher rates in Sudan and Egypt (44.3%, and 41,4% respectively). While the prevalence of FD in these 2 countries has not been studied before, the pooled prevalence of FD in African countries in general in 2014 was 35.7%.^[2]^ This means that we are witnessing a significant increase in the spread of the disease in Africa.

As the disease was more prevalent among the Afro-Caribbean race (47.9%). This suggests the influence of genetic factors on the emergence of the disease, in addition to the habits and cultures of peoples. Asian FD prevalence was 28.1%; This percentage is slightly higher than that reported in a review published in 2011 (23%).^[9]^ However, variation in the methods used to diagnose the disease led to variation in prevalence rates among the different studies.^[10]^

Our study is the first to investigate the prevalence of the disease in countries such as Syria, Egypt, Sudan, Algeria and Pakistan, and it is the first to determine the prevalence among Caucasians in general.

### 4.2. Non-modifiable risk factors

Several factors are associated with a higher probability of FD. The female gender was a risk factor for FD. This can be justified by the differences in sex hormones, which mainly affect the main factor characteristic of FD, GI motility.^[2,11]^ In addition, hormonal changes during the menstrual cycle are associated with visceral pain. The other distinguishing factor of FD.^[12]^ All of the above explains the fact that females are more susceptible to the disease than males.

Advanced age is also among the most important non-modifiable risk factors that have been associated with higher rates of disease. This was confirmed by a Korean multicenter prospective study,^[13]^ in which disease risk factors were studied separately according to age and gender. It is still difficult to know why the disease is associated with people of advanced ages. The Korean study, for example, attributed the reason to the high prevalence of the psychological co-morbidity in the elderly. However, we see that answering this question still requires a greater understanding of the molecular mechanism of this disease.

Non-modifiable risk factors also include mild and severe systemic diseases. The presence of the disease has been associated with certain chronic systemic diseases, such as a personal^[14]^ history of chronic diseases (especially hypertension and diabetes) and autoimmune diseases. Perhaps this association can be explained from an immunological point of view. Considering the immune activation following loss of mucosal integrity in the course of FD.^[10,15]^ The disease has also been previously linked to many other chronic systemic diseases, such as chronic hepatitis C.^[14]^

### 4.3. Modifiable risk factors

Regarding the modifiable risk factors, smoking is one of the most important. This effect of smoking can be explained by the fact that tobacco changes the duodenal microbiome, which plays a major role in the pathogenesis of FD.^[16,17]^ A global meta-analysis of 9 studies found a higher prevalence of FD among smokers.

Modifiable risk factors also included abnormal BMI (whether high or low). A cross-sectional study of 1002 young adults from Malaysia found a higher prevalence of the disease among the underweight.^[18]^ This was confirmed by the results of a similar Japanese study.^[2]^ While another study from Korea found an association of FD with visceral adiposity.^[19]^ However, our study shows that the disease is associated with high or low abnormal BMI.

Regarding diet, several studies have indicated the potential effect of coffee on dyspepsia. Also, coffee increases the production of gastric acid.^[20,21]^ Whereas carbonated beverages decrease dyspepsia and improve gastric and intestinal function.^[22]^

Our study shows a significant increase in FD prevalence in the participants with high stress compared to moderate and low stress. There is a significant relationship between depression, anxiety, and FD.^[23–25]^ In a study of patients with dyspepsia, and by using of the Depression, Anxiety and Stress scale (DASS), 67.5% of patients had stress, 60% depression, and 82.5% were found to have anxiety.^[26]^

Some fields of jobs and living in a rural area are also modifiable risk factors for FD. However, in our study, physicians were less likely to be patients than other professions, this could be due to doctors’ knowledge of the etiology and preventive ways of this disease, however, more studies are needed on this aspect.

### 4.4. Clinical implications

Here are some highlights of the clinical implications and potential strategies based on the findings from this cross-sectional study on FD prevalence and risk factors:

Clinical implications:

- The high FD prevalence (25.2%) demonstrates this is a major chronic digestive disease burden in developing countries that needs focused attention.
- Positive associations with modifiable factors like diet, smoking, activity levels, etc underscore the potential for lifestyle management.
- Protective effect seen in healthcare workers reinforces the value of patient education and awareness.

Potential strategies:

- Increasing FD awareness and screening practices in primary care in developing countries to improve early diagnosis.
- Lifestyle counseling by dieticians, social workers and community health workers on diet, activity, sleep hygiene.
- Smoking cessation programs and resources for patients with FD where relevant.
- Self-management workshops on stress reduction techniques like yoga, meditation, cognitive behavioral therapies for FD patients.
- Culturally appropriate educational campaigns on FD using diverse platforms to reach rural areas.
- Policy efforts to promote physical activity through built environment changes in developing country urban areas.

In summary, the high disease burden calls for greater health system prioritization and lifestyle-based management with tailored sociocultural approaches to curb preventable risk factors.

### 4.5. Potential confounding variables

- *Age*: FD prevalence increased with age. Age may confound associations between other factors and FD.
- *Gender*: Females had higher FD prevalence. Gender differences could confound other observed relationships.
- *Comorbidities*: Chronic diseases like diabetes and hypertension were associated with higher FD prevalence. These may confound other variables.
- *Medications*: Drugs used for comorbid conditions could influence FD risk, confounding associations.
- *Diet*: Dietary patterns like high fat/protein diet were associated with FD. Diet may be a confounder.
- *Physical activity*: Lack of adequate activity was linked to higher FD prevalence. This could confound other observations especially in the outcomes of certain jobs.

### 4.6. Limitations of the study

Our study included countries did not have sufficient data regarding FD prevalence before. Nonetheless, there were some limitations despite the variety of nationalities included in the study, half of the population was from Syria. We tried to overcome this by including participants from different geographical areas and different socioeconomic backgrounds. Also, most of the population was Caucasian; other ethnicities were represented by much fewer ratios. This is because Caucasian is the predominant ethnicity in the included countries. Finally, our study is cross-sectional; we were able to study the association between several factors and FD. However, this cross-sectional study may reflect the FD prevalence and risk factors in low- and middle-income countries. Individuals living in high-income countries were not included in this study.

## 5. Conclusion

Our study concluded that the prevalence of FD is much higher in low- and middle-income countries 37.9% [6.2%–44.2%], compared to high-income countries 10% [8%–12%]. Moreover, there are many risk factors, including modifiable ones, such as severe stress, chronic fatigue, smoking, abnormal BMI, insufficient or too many hours of sleep, and previous infection with Covid-19, and non-modifiable ones such as advanced age, chronic diseases, and female sex.

## Author contributions

**Conceptualization:** Ahmad Yamen Arnaout, Khaled Arnaout, Ziad aljarad.

**Data curation:** Ahmad Yamen Arnaout, Ziad aljarad.

**Formal analysis:** Ahmad Yamen Arnaout.

**Methodology:** Ahmad Yamen Arnaout.

**Project administration:** Ahmad Yamen Arnaout, Ziad aljarad.

**Supervision:** Khaled Arnaout, Ziad aljarad.

**Validation:** Ahmad Yamen Arnaout, Yaman Nerabani, Khaled Arnaout, Ziad aljarad.

**Visualization:** Tala Jouma Alhejazi, Yaman Nerabani.

**Writing – original draft:** Ahmad Yamen Arnaout, Tala Jouma Alhejazi, Yaman Nerabani, Ola Hamdan, Ibrahim Arnaout.

**Writing – review & editing:** Ahmad Yamen Arnaout, Tala Jouma Alhejazi, Yaman Nerabani, Ola Hamdan, Khaled Arnaout, Ibrahim Arnaout.

## Supplementary Material
