# Supplementary material for: The prevalence and risk factors of functional dyspepsia among adults in low- and middle-income countries: An international cross-sectional study
Source: Medicine (Baltimore). 2023 Oct 6;102(40):e35437. doi: 10.1097/MD.0000000000035437 (PMC10553146; doi:10.1097/MD.0000000000035437)
Supplement: Supplementary file 1 [file medi-102-e35437-s001.docx]

**Co-Authors for PRIBS Study Team**

| **Country** | **Co-Author Name** | **E-mail** | **ORCID** | **Task** |
| --- | --- | --- | --- | --- |
| **Syria** | **Ahmad Yousef** | **ahmadyusuf1233210@gmail.com** | **0000-0002-5075-1094** | **National Lead** |
| **Syria** | **Abd Alazeez Atli** | **abdalazizatli@gmail.com** | **0000-0001-7951-3869** | **Data Collection** |
| **Syria** | **Ahmad Chehabi** | [**dodishehabi@hotmail.com**](mailto:dodishehabi@hotmail.com) | **0000-0003-4181-8282** | **Data Collection** |
| **Syria** | **Ahmad Haj Asaad** | **ahmadhajasaad999@gmail.com** | **0000-0001-5926-7461** | **Data Collection** |
| **Syria** | **Ahmad Hanino** | **www.ahmadhanino2@gmail.com** | **0000-0001-9382-2238** | **Data Collection** |
| **Syria** | **Ahmad Khaled** | **ak8213886@gmail.com** | **0000-0002-6881-0005** | **Data Collection** |
| **Syria** | **Ahmad Nahawi** | **Ahmdnhwy136@gmail.com** | **0000-0001-8622-3819** | **Data Collection** |
| **Syria** | **Alaa Hawarah** | **alaa.mha19@gmail.com** | **0000-0002-2605-8070** | **Data Collection** |
| **Syria** | **Alhasan Alkhayer** | **alhasan.alkhayer9@gmail.com** | **0000-0002-3197-7231** | **Data Collection** |
| **Syria** | **Ali Abbas** | **aliasaadabbas.97@gmail.com** | **0000-0002-8023-3992** | **Data Collection** |
| **Syria** | **Ali Mahmoud Ahmad** | **Ali.0932820336@gmail.com** | **0000-0002-1881-8754** | **Data Collection** |
| **Syria** | **Amana Kezze** | **amana.k6be.careful@gmail.com** | **0000-0003-1683-9241** | **Data Collection** |
| **Syria** | **Ameena Odeh** | **ameena.odeh@hotmail.com** | **0000-0002-5401-8041** | **Data Collection** |
| **Syria** | **Bashar Bazkke** | **bashar.bazkke@gmail.com** | **0000-0001-6097-9220** | **Data Collection** |
| **Syria** | **Besher Alhadi Asadi** | **Besher.asadi@gmail.com** | **0000-0002-3760-5551** | **Data Collection** |
| **Syria** | **Danya Mourad** | **danyamourad@gmail.com** | **0000-0003-3541-0205** | **Data Collection** |
| **Syria** | **Elissa abd elfattah** | **smile4ever691998@gmail.com** | **0000-0001-5126-0804** | **Data Collection** |
| **Syria** | **Esraa Jlelatie** | **esraa.jlelatie@gmail.com** | **0000-0002-6862-3920** | **Data Collection** |
| **Syria** | **Ezeddin Dabbagh** | **ezzalldin.da@gmail.com** | **0000-0002-1229-8380** | **Data Collection** |
| **Syria** | **Fahima Taleb** | **fahimataleb62@gmail.com** | **0000-0002-2075-9239** | **Data Collection** |
| **Syria** | **Ghina Jamil Majed** | **jamilmajid2016@hotmail.com** | **0000-0002-3906-3776** | **Data Collection** |
| **Syria** | **Haidar Barakat** | **haidar0barakat@gmail.com** | **0000-0003-0017-4679** | **Data Collection** |
| **Syria** | **Halla Barri** | **barrihalla@gmail.com** | **0000-0003-0167-2960** | **Data Collection** |
| **Syria** | **Hasan Ali Maroush** | **hassan.marouch1998@gmail.com** | **0000-0001-8313-9283** | **Data Collection** |
| **Syria** | **Hayat al. Agha** | **hayat.a.alagha@gmail.com** | **0000-0002-2276-7005** | **Data Collection** |
| **Syria** | **Heba Cheikh Othman** | **hebashekhothman@gmail.com** | **0000-0002-9727-9719** | **Data Collection** |
| **Syria** | **Hiba Allah Sarraj** | **www.hibasarraj@gmail.com** | **0000-0002-9159-9148** | **Data Collection** |
| **Syria** | **Ibrahim Khezaran** | **ibrahim.khezaran98@gmail.com** | **0000-0001-8351-0994** | **Data Collection** |
| **Syria** | **Jaber Baki Zada** | **jaber.b.k.z.q.q@gmail.com** | **0000-0001-6442-6973** | **Data Collection** |
| **Syria** | **Joudy Om Alola Sheet** | **joudy.sheet@gmail.com** | **0000-0003-0192-4654** | **Data Collection** |
| **Syria** | **Lana Almahairi** | **lanaalmahairy@gmail.com** | **0000-0003-2308-9928** | **Data Collection** |
| **Syria** | **Layal Alshiekh** | **alsheikhlayal@gmail.com** | **0000-0002-9401-5451** | **Data Collection** |
| **Syria** | **Maen Al-Najjar** | **maen.alnajjar2860@gmail.com** | **0000-0002-7658-0561** | **Data Collection** |
| **Syria** | **Mahmoud Koritbi** | **mahmoudkoritby@gmail.com** | **0000-0001-6031-312X** | **Data Collection** |
| **Syria** | **Majd-Aldin Sabhan** | **majood.m.sbhan@gmail.com** | **0000-0003-1814-3766** | **Data Collection** |
| **Syria** | **Marah Khalil** | **marooha.99@gmail.com** | **0000-0002-0702-449X** | **Data Collection** |
| **Syria** | **Mawya Alrawi** | **mawya2001alrawi@gmail.com** | **0000-0003-1544-2306** | **Data Collection** |
| **Syria** | **Mofida Ghannam** | **mofidaghannam00@gmail.com** | **0000-0002-2428-7005** | **Data Collection** |
| **Syria** | **Mohamad Ali Farho** | **ali_fa_2001@hotmail.com** | **0000-0002-3277-2270** | **Data Collection** |
| **Syria** | **Mohamad Al-mahdi Al-kurdi** | **mohammedmahdikurdi@gmail.com** | **0000-0002-1858-8450** | **Data Collection** |
| **Syria** | **Mohamad Nabhan Sawas** | **mo.nabhan.sa@gmail.com** | **0000-0003-4060-6755** | **Data Collection** |
| **Syria** | **Mohamad Sbeinati** | **mohamad.sbeinati@gmail.com** | **0000-0001-8308-3590** | **Data Collection** |
| **Syria** | **Mohamad Shehab Alyousfi** | **mohammadshehabalyousfi@gmail.com** | **0000-0002-0005-0898** | **Data Collection** |
| **Syria** | **Mohammad Ahmad Faksh** | **mohammadfaksh658@gmail.com** | **0000-0001-8378-8021** | **Data Collection** |
| **Syria** | **Mohammad ali alkouje** | **mohamed.alkouje@gmail.com** | **0000-0002-5062-5100** | **Data Collection** |
| **Syria** | **Mohammad Alkhawalda** | **mjkhawaldeh@gmail.com** | **0000-0001-7260-7707** | **Data Collection** |
| **Syria** | **Mohammad Shahrour** | [**mohammadshahrour21@gmail.com**](mailto:mohammadshahrour21@gmail.com) | **0000-0001-7506-9924** | **Data Collection** |
| **Syria** | **Mohanad Daher** | **muhanaddaher8@gmail.com** | **0000-0002-0617-6280** | **Data Collection** |
| **Syria** | **Monzer keblawy** | **monzerkeblawi@gmail.com** | **0000-0002-9075-2446** | **Data Collection** |
| **Syria** | **Nour Halwani** | **nourhalwanii99@gmail.com** | **0000-0002-3025-4330** | **Data Collection** |
| **Syria** | **Nour Jreikh** | **nour2000j1@gmail.com** | **0000-0001-6987-0635** | **Data Collection** |
| **Syria** | **Ola Alkhallouf** | **olakhallouf472@gmail.com** | **0000-0002-4657-9666** | **Data Collection** |
| **Syria** | **Omar Najjar** | **omarnajjar934@gmail.com** | **0000-0002-0488-9826** | **Data Collection** |
| **Syria** | **Qusai Razzouk** | **qusayr2000@gmail.com** | **0000-0002-9540-1100** | **Data Collection** |
| **Syria** | **Raghad Sawas** | **raghadsawas96@gmail.com** | **0000-0001-8122-533X** | **Data Collection** |
| **Syria** | **Rama Aboudan** | **rama.11aboudan@gmail.com** | **0000-0002-1618-7071** | **Data Collection** |
| **Syria** | **Rama Summak** | **RSummak@gmail.com** | **0000-0002-1960-1135** | **Data Collection** |
| **Syria** | **Rama Zannerni** | **ramazannerni3@gmail.com** | **0000-0001-8908-8966** | **Data Collection** |
| **Syria** | **Ramez Shahin** | **ramezshahin4@gmail.com** | **0000-0001-8854-3453** | **Data Collection** |
| **Syria** | **Rand Ibrahim** | **roroibrahim1722@gmail.com** | **0000-0002-4415-2060** | **Data Collection** |
| **Syria** | **Razan hajjouz** | **razanhajjouz1998@gmail.com** | **0000-0001-8084-7495** | **Data Collection** |
| **Syria** | **Saffana krayem** | **Saffana.krayem99@gmail.com** | **0000-0002-0066-593X** | **Data Collection** |
| **Syria** | **Sally Korini** | **s.0958371433@gmail.com** | **0000-0001-5329-8240** | **Data Collection** |
| **Syria** | **Sami abedalkader Abedalkader** | **samialmofty.7@gmail.com** | **0000-0002-0785-2770** | **Data Collection** |
| **Syria** | **Sana Oubari** | **sanaoubari1999@gmail.com** | **0000-0003-2342-9734** | **Data Collection** |
| **Syria** | **Sanaa Nahhas** | **sanaanahhas@gmail.com** | **0000-0003-3180-3984** | **Data Collection** |
| **Syria** | **Sedra Kreid** | **Sidra.kd.2000@gmail.com** | **0000-0003-4043-3436** | **Data Collection** |
| **Syria** | **Shahd Maan Alamoura** | **shahed.maam@gmail.com** | **0000-0002-4889-2525** | **Data Collection** |
| **Syria** | **Shahd Maarrawi** | **shahdmaarrawi@gmail.com** | **0000-0002-4075-5526** | **Data Collection** |
| **Syria** | **Shahd Merhej** | **Shahdmerhej2002@gmail.com** | **0000-0002-2521-7623** | **Data Collection** |
| **Syria** | **Sherine hagi shamou** | **sherinecomputer@gmail.com** | **0000-0003-0643-7142** | **Data Collection** |
| **Syria** | **Siham Alabrash** | **sihamalabrash@gmail.com** | **0000-0002-2060-6056** | **Data Collection** |
| **Syria** | **Somar Berro** | **somerberro@gmail.com** | **0000-0001-6443-9408** | **Data Collection** |
| **Syria** | **Tala Jouma Alhejazi** | **talahij1432@gmail.com** | **0000-0001-6272-4184** | **Data Collection** |
| **Syria** | **Turfa Moudarres** | **turfaaa99@gmail.com** | **0000-0003-1519-4859** | **Data Collection** |
| **Syria** | **Walaa Qirata** | **walaaqirata@gmail.com** | **0000-0001-8533-4621** | **Data Collection** |
| **Syria** | **Yahya Smadi** | **yahyasamady.1997@gmail.com** | **0000-0002-8660-9313** | **Data Collection** |
| **Syria** | **Yasmeen saeed rajab** | **yasmynrjb66@gmail.com** | **0000-0003-0612-7857** | **Data Collection** |
| **Syria** | **Youmen Srajaldeen** | **yomensrajaldeen@gmail.com** | **0000-0002-2988-8416** | **Data Collection** |
| **Syria** | **Rami Anadani** | **ramianadani99@gmail.com** | **0000-0001-5461-4241** | **Data Collection** |
| **Syria** | **Reem Kozum** | **reem.kozum.1998@gmail.com** | **0000-0002-8143-9140** | **Data Collection** |
| **Syria** | **Othman Sheikh Hussein** | **othmanshiekhhussain@gamil.com** | **0000-0002-8228-7898** | **Data Collection** |
| **Egypt** | **Mustafa Alsebaei** | **mustafaalsebaei08@gmail.com** | **0000-0001-7250-5428** | **Data Collection** |
| **Egypt** | **Abdelrahman Shawky Refaee** | **abdorefaee@yahoo.com** | **0000-0002-1981-9425** | **Data Collection/ National Lead** |
| **Egypt** | **Albaraa Daradkeh** | **elbaraa.mahmoud1901@alexmed.edu.eg** | **0000-0002-8282-8236** | **Data Collection** |
| **Egypt** | **Alshaymaa Mortada Ali Eltohry** | **alshaymaaali62@gmail.com** | **0000-0001-6867-8326** | **Data Collection** |
| **Egypt** | **Azza Osama abdelmetaal alqurei** | **azzausama47@gmail.com** | **0000-0002-5436-8818** | **Data Collection** |
| **Egypt** | **Donia Amgad Farhat** | **donia_farhat@yahoo.com** | **0000-0003-1379-1908** | **Data Collection** |
| **Egypt** | **Eslam Mohamad Elshennawy** | **eslam_alshenawy@med.kfs.edu.eg** | **0000-0003-1410-7784** | **Data Collection** |
| **Egypt** | **Fatma Yousef abdelaziz abo elnaga** | **batayousef03@gmail.com** | **0000-0002-9534-5444** | **Data Collection** |
| **Egypt** | **Ghada Mahmoud Hussien Eid** | **ghadamahmoud088@gmail.com** | **0000-0002-7606-4364** | **Data Collection** |
| **Egypt** | **Marwa Mahmoud Soliman Sabaa** | **marwa_sabaa@yahoo.com** | **0000-0003-3885-0943** | **Data Collection** |
| **Egypt** | **Mo’men Mohamed Roshdy** | **moemenroshdy2016@gmail.com** | **0000-0002-3803-0817** | **Data Collection** |
| **Egypt** | **mohamed said abdelkader koreitam** | **m_korietum@yahoo.com** | **0000-0003-0342-9550** | **Data Collection** |
| **Egypt** | **Neamt Mahmoud Amin Hassan sakr** | **Neamtsakr@yahoo.com** |  | **Data Collection** |
| **Egypt** | **Radwa Eletr** | **radwa.atef41@gmail.com** | **0000-0002-5040-2417** | **Data Collection** |
| **Pakistan** | **Abdul Basit** | **abdulbasitrauf97@gmail.com** | **0000-0003-2189-080X** | **Data Collection** |
| **Pakistan** | **Aleena Batool** | **aleenabatool114@gmail.com** | **0000-0001-6235-3643** | **Data Collection** |
| **Pakistan** | **Anshahrah Riaz** | **anshahrahriaz8@gmail.com** | **0000-0002-4568-7307** | **Data Collection** |
| **Pakistan** | **Aqsa Iqbal** | **aqsaiqbal965@gmail.com** | **0000-0003-4719-6058** | **Data Collection** |
| **Pakistan** | **Ibad ur Rehman** | **ibadrehmaan@gmail.com** | **0000-0001-9639-0741** | **Data Collection** |
| **Pakistan** | **Izma Ajaz** | **izmaajaz@gmail.com** | **0000-0002-6287-0031** | **Data Collection** |
| **Pakistan** | **Namrah Anwer** | **nemo_anwer@outlook.com** | **0000-0003-4374-3843** | **Data Collection** |
| **Pakistan** | **Syeda Tahira Waheed** | **tahirahashmi20@gmail.com** | **0000-0002-1478-1309** | **Data Collection** |
| **Pakistan** | **Warda khan** | **warda.khan4902@gmail.com** | **0000-0002-3104-1433** | **Data Collection** |
| **Sudan** | **ABOBAKR ABDALLAH MOHAMED OSMAN** | **abobakr97abdallah11@gmail.com** | **0000-0002-3400-6561** | **Data Collection** |
| **Sudan** | **Abubakr Elsadig Musa Muhammed** | **abubakr35007@gmail.com** | **0000-0003-2658-000X** | **Data Collection** |
| **Sudan** | **Albushra Altayeb Adam Osman** | **albushraaltayeb@gmail.com** | **0000-0001-6594-8730** | **Data Collection** |
| **Sudan** | **Asjad Hassan Eltayeb Shamseldeen** | **jody.hassan26@gmail.com** | **0000-0002-7486-3912** | **Data Collection** |
| **Sudan** | **Esraa Hammad AbdAllah Ageeb** | **toriageeb302@gmail.com** | **0000-0002-5376-8465** | **Data Collection** |
| **Sudan** | **Fatima Mohamed Awad Osman** | **fatmo4160@gmail.com** | **0000-0002-1563-7296** | **Data Collection** |
| **Sudan** | **Maysoon Nagmeldin Mursi Mohamedshafee** | **maysoony99@gmail.com** | **0000-0001-6273-8543** | **Data Collection** |
| **Sudan** | **Moaz Noureldin Easa El Tayeb** | **moaznoreldeen@gmail.com** | **0000-0002-5821-295X** | **Data Collection** |
| **Sudan** | **Mohamed Alghazali** | **mhghazally@gmail.com** | **0000-0002-5465-5366** | **Data Collection** |
| **Sudan** | **Mohamed hassan Tagelkhatim Mohamed** | **mo7.alves1994@hotmail.com** | **0000-0003-2097-4585** | **Data Collection** |
| **Sudan** | **Rawnag Ali Mubarak Ali** | **www.rawnag888@gmail.com** | **0000-0002-5940-5155** | **Data Collection** |
| **Sudan** | **Sarah Ahmed Alhassan** | **saraosama797@gmail.com** | **0000-0002-4813-0539** | **Data Collection** |
| **Sudan** | **Taqwa Elyas Altahir Alshaikh** | **taqwaleyas@gmail.com** | **0000-0001-6811-3456** | **Data Collection** |
| **Libya** | **Ayman abulrassul Hasan aboulqassim** | **alsharifayman1986@gmail.com** | **0000-0001-6244-8618** | **Data Collection** |
| **Libya** | **Eshrak Saad Ahmed** | **Splendoure94@gmail.com** | **0000-0002-1486-0226** | **Data Collection/ National Lead** |
| **Libya** | **Huda Ahmed Muftah Aqmati** | **hudaaqmati852@gmail.com** | **0000-0001-6988-8253** | **Data Collection** |
| **Libya** | **Khwla F.Magid** | **khwlafadil34@gmail.com** | **0000-0002-5189-0088** | **Data Collection** |
| **Libya** | **Moufiq abdulrasoul Hasan aboulqassim** | **mowfagoz@gmail.com** | **0000-0002-8015-8611** | **Data Collection** |
| **Libya** | **Zinelabedin Mohamed** | **Zen_zen47@yahoo.com** | **0000-0003-0133-809X** | **Data Collection/ National Lead** |
| **Libya** | **Najat Ahmed Hashem Mohammed** | **najatahmed003@gmail.com** |  | **Data Collection** |
| **Algeria** | **Rais Mohammed Amir** | **raismohammedamir@gmail.com** | **0000-0002-1290-4379** | **Data Collection/ National Lead** |
| **Algeria** | **Assia Salah** | **assia-salah@outlook.fr** | **0000-0003-0619-5243** | **Data Collection** |
| **Algeria** | **Djedidi Lamis** | **djedidilamis@gmail.com** | **0000-0002-6162-8868** | **Data Collection** |
| **Algeria** | **Ihcene Gourari** | **contact.ihcene@gmail.com** | **0000-0001-8900-9527** | **Data Collection** |
| **Algeria** | **Manare kahoul** | **kahoulmanare@gmail.com** | **0000-0003-3780-8292** | **Data Collection** |
| **Algeria** | **Rayane Dorbane** | **m20414@univ-constantine3.dz** | **0000-0001-5618-2366** | **Data Collection** |
| **Algeria** | **ABDERRAZAK MOHAMMED** | **med.abderrazak@yahoo.com** | **0000-0003-4474-1292** | **Data Collection** |
| **Jordan** | **Ahmad Alshkirat** | **ahm0190145@ju.edu.jo** | **0000-0002-7005-4229** | **Data Collection** |
| **Jordan** | **Ghalib Nashaat El Hunjul** | **ghalibhunjul@gmail.com** | **0000-0002-4439-7519** | **Data Collection** |
| **Jordan** | **Hashem Altabbaa** | **hashem0908@hotmail.com** | **0000-0002-8031-838X** | **Data Collection** |
| **Jordan** | **Laith Shakhatreh** | **laithshakhatreh99@gmail.com** | **0000-0002-9060-3942** | **Data Collection** |
| **Jordan** | **Mo'ath Al-Hazaimeh** | **spaceking1999@gmail.com** | **0000-0002-9666-7808** | **Data Collection** |
| **Jordan** | **Sereen khasawneh** | **sereen-2000@hotmail.com** | **0000-0002-8503-6218** | **Data Collection** |
| **India** | **Dhruv Tewari** | **bukshukchin2@gmail.com** | **0000-0001-5886-8089** | **Data Collection** |
| **India** | **Rachana Reddy Dasireddy** | **rachanadasireddyksrr@gmail.com** | **0000-0001-6154-9134** | **Data Collection** |
| **India** | **Rishabh Kapil** | **kapilrishabh09@gmail.com** | **0000-0001-6999-489X** | **Data Collection** |
| **Yemen** | **Dua Hassan Hassan Mohammed Abu-Ali** | **doctorduaa331@gmail.com** | **0000-0003-0174-7782** | **Data Collection** |
| **Yemen** | **Abdulghani Ahmed Ali Al-Aswadi** | **abdulghanialaswadi1@gmail.com** | **0000-0001-7469-8606** | **Data Collection** |
| **Saudi Arabia** | **Ahmed Mohamed** | **AHMADALFADEL777@GMAIL.COM** | **0000-0001-5996-7097** | **Data Collection** |
| **United Arab Emirates** | **Haya Mohammed Zakaria Mashhadi** | **Hayamzm@gmail.com** | **0000-0002-5480-478X** | **Data Collection** |
| **Morocco** | **Boukhiam Meriem** | **boukhiameriem@gmail.com** | **0000-0001-5828-1456** | **Data Collection** |
| **Palestine** | **Bashar Mohamed AlHennawi** | **bashar.hennawi@gmail.com** | **0000-0001-9357-6233** | **Data Collection** |
| **Iraq** | **Israa Abduljaleel Al-fayyadh** | **israa.med@hotmail.com** | **0000-0003-2352-3771** | **Data Collection** |
| **Serbia** | **Abd Alrazak Albasis** | [**abdulrazzakalbasis99@gmail.com**](mailto:abdulrazzakalbasis99@gmail.com) | **0000-0003-0943-4051** | **Data Collection** |
| **Serbia** | **ABDALMOEEN YOUSEF ALBASIS** | **Aboodalbasis@gmail.com** | **0000-0001-7155-5853** | **Data Collection** |
