# Supplementary material for: The prevalence and risk factors of functional dyspepsia among adults in low- and middle-income countries: An international cross-sectional study
Source: Medicine (Baltimore). 2023 Oct 6;102(40):e35437. doi: 10.1097/MD.0000000000035437 (PMC10553146; doi:10.1097/MD.0000000000035437)
Supplement: Supplementary file 2 [file medi-102-e35437-s002.docx]

Sub-Groups Analysis (Post Prandial Distress Syndrome, Epigastric Pain Syndrome)

**Crosstabs**

| **Notes** | | |
| --- | --- | --- |
| Output Created | | 24-FEB-2023 15:00:17 |
| Comments | |  |
| Input | Active Dataset | DataSet1 |
|  | Filter | <none> |
|  | Weight | <none> |
|  | Split File | <none> |
|  | N of Rows in Working Data File | 5505 |
| Missing Value Handling | Definition of Missing | User-defined missing values are treated as missing. |
|  | Cases Used | Statistics for each table are based on all the cases with valid data in the specified range(s) for all variables in each table. |

| **Case Processing Summary** | | | | | | |
| --- | --- | --- | --- | --- | --- | --- |
|  | Cases | | | | | |
|  | Valid | | Missing | | Total | |
|  | N | Percent | N | Percent | N | Percent |
| Epigastric pain syndrome (EPS) * Sex | 5505 | 100.0% | 0 | 0.0% | 5505 | 100.0% |
| Epigastric pain syndrome (EPS) * Ethnicity | 5505 | 100.0% | 0 | 0.0% | 5505 | 100.0% |
| Epigastric pain syndrome (EPS) * ASA Grade | 5505 | 100.0% | 0 | 0.0% | 5505 | 100.0% |
| Epigastric pain syndrome (EPS) * Geographic | 5505 | 100.0% | 0 | 0.0% | 5505 | 100.0% |
| Epigastric pain syndrome (EPS) * Hypertension requiring medication | 5505 | 100.0% | 0 | 0.0% | 5505 | 100.0% |
| Epigastric pain syndrome (EPS) * Diabetes Mellitus | 5505 | 100.0% | 0 | 0.0% | 5505 | 100.0% |
| Epigastric pain syndrome (EPS) * Autoimmune diseases | 5505 | 100.0% | 0 | 0.0% | 5505 | 100.0% |
| Epigastric pain syndrome (EPS) * Headache or migraine | 5505 | 100.0% | 0 | 0.0% | 5505 | 100.0% |
| Epigastric pain syndrome (EPS) * Chronic immunosuppression | 5505 | 100.0% | 0 | 0.0% | 5505 | 100.0% |
| Epigastric pain syndrome (EPS) * Anemia | 5505 | 100.0% | 0 | 0.0% | 5505 | 100.0% |
| Epigastric pain syndrome (EPS) * Patients allergic to certain substances | 5505 | 100.0% | 0 | 0.0% | 5505 | 100.0% |
| Epigastric pain syndrome (EPS) * Chronic obstructive pulmonary disease (COPD) | 5505 | 100.0% | 0 | 0.0% | 5505 | 100.0% |
| Epigastric pain syndrome (EPS) * COVID-19 infection | 5505 | 100.0% | 0 | 0.0% | 5505 | 100.0% |
| Epigastric pain syndrome (EPS) * Open abdominal surgery/laparotomy | 5505 | 100.0% | 0 | 0.0% | 5505 | 100.0% |
| Epigastric pain syndrome (EPS) * (CFQ) Scale | 5505 | 100.0% | 0 | 0.0% | 5505 | 100.0% |
| Epigastric pain syndrome (EPS) * Smoking | 5505 | 100.0% | 0 | 0.0% | 5505 | 100.0% |
| Epigastric pain syndrome (EPS) * Alcohol | 5505 | 100.0% | 0 | 0.0% | 5505 | 100.0% |
| Epigastric pain syndrome (EPS) * PSS Scale | 5505 | 100.0% | 0 | 0.0% | 5505 | 100.0% |
| Postprandial distress syndrome (PPDS) * Sex | 5505 | 100.0% | 0 | 0.0% | 5505 | 100.0% |
| Postprandial distress syndrome (PPDS) * Ethnicity | 5505 | 100.0% | 0 | 0.0% | 5505 | 100.0% |
| Postprandial distress syndrome (PPDS) * ASA Grade | 5505 | 100.0% | 0 | 0.0% | 5505 | 100.0% |
| Postprandial distress syndrome (PPDS) * Geographic | 5505 | 100.0% | 0 | 0.0% | 5505 | 100.0% |
| Postprandial distress syndrome (PPDS) * Hypertension requiring medication | 5505 | 100.0% | 0 | 0.0% | 5505 | 100.0% |
| Postprandial distress syndrome (PPDS) * Diabetes Mellitus | 5505 | 100.0% | 0 | 0.0% | 5505 | 100.0% |
| Postprandial distress syndrome (PPDS) * Autoimmune diseases | 5505 | 100.0% | 0 | 0.0% | 5505 | 100.0% |
| Postprandial distress syndrome (PPDS) * Headache or migraine | 5505 | 100.0% | 0 | 0.0% | 5505 | 100.0% |
| Postprandial distress syndrome (PPDS) * Chronic immunosuppression | 5505 | 100.0% | 0 | 0.0% | 5505 | 100.0% |
| Postprandial distress syndrome (PPDS) * Anemia | 5505 | 100.0% | 0 | 0.0% | 5505 | 100.0% |
| Postprandial distress syndrome (PPDS) * Patients allergic to certain substances | 5505 | 100.0% | 0 | 0.0% | 5505 | 100.0% |
| Postprandial distress syndrome (PPDS) * Chronic obstructive pulmonary disease (COPD) | 5505 | 100.0% | 0 | 0.0% | 5505 | 100.0% |
| Postprandial distress syndrome (PPDS) * COVID-19 infection | 5505 | 100.0% | 0 | 0.0% | 5505 | 100.0% |
| Postprandial distress syndrome (PPDS) * Open abdominal surgery/laparotomy | 5505 | 100.0% | 0 | 0.0% | 5505 | 100.0% |
| Postprandial distress syndrome (PPDS) * (CFQ) Scale | 5505 | 100.0% | 0 | 0.0% | 5505 | 100.0% |
| Postprandial distress syndrome (PPDS) * Smoking | 5505 | 100.0% | 0 | 0.0% | 5505 | 100.0% |
| Postprandial distress syndrome (PPDS) * Alcohol | 5505 | 100.0% | 0 | 0.0% | 5505 | 100.0% |
| Postprandial distress syndrome (PPDS) * PSS Scale | 5505 | 100.0% | 0 | 0.0% | 5505 | 100.0% |

**Epigastric pain syndrome (EPS) * Sex**

| **Crosstab** | | | | | |
| --- | --- | --- | --- | --- | --- |
|  | | | Sex | | Total |
|  |  |  | Female | Male |  |
| Epigastric pain syndrome (EPS) | 0 | Count | 2297 | 1765 | 4062 |
|  |  | % within Epigastric pain syndrome (EPS) | 56.5% | 43.5% | 100.0% |
|  |  | % within Sex | 71.7% | 76.7% | 73.8% |
|  |  | % of Total | 41.7% | 32.1% | 73.8% |
|  | 1 | Count | 907 | 536 | 1443 |
|  |  | % within Epigastric pain syndrome (EPS) | 62.9% | 37.1% | 100.0% |
|  |  | % within Sex | 28.3% | 23.3% | 26.2% |
|  |  | % of Total | 16.5% | 9.7% | 26.2% |
| Total | | Count | 3204 | 2301 | 5505 |
|  |  | % within Epigastric pain syndrome (EPS) | 58.2% | 41.8% | 100.0% |
|  |  | % within Sex | 100.0% | 100.0% | 100.0% |
|  |  | % of Total | 58.2% | 41.8% | 100.0% |

| **Chi-Square Tests** | | | | | |
| --- | --- | --- | --- | --- | --- |
|  | Value | df | Asymptotic Significance (2-sided) | Exact Sig. (2-sided) | Exact Sig. (1-sided) |
| Pearson Chi-Square | 17.408^a^ | 1 | .000 |  |  |
| Continuity Correction^b^ | 17.150 | 1 | .000 |  |  |
| Likelihood Ratio | 17.549 | 1 | .000 |  |  |
| Fisher's Exact Test |  |  |  | .000 | .000 |
| N of Valid Cases | 5505 |  |  |  |  |
| a. 0 cells (0.0%) have expected count less than 5. The minimum expected count is 603.15. | | | | | |
| b. Computed only for a 2x2 table | | | | | |

| **Symmetric Measures^a^** | |
| --- | --- |
|  | Value |
| N of Valid Cases | 5505 |
| a. Correlation statistics are available for numeric data only. | |

**Epigastric pain syndrome (EPS) * Ethnicity**

| **Crosstab** | | | | | | | | |
| --- | --- | --- | --- | --- | --- | --- | --- | --- |
|  | | | Ethnicity | | | | | Total |
|  |  |  | Afro-Caribbean | Asian | Caucasian | Hispanic | Other |  |
| Epigastric pain syndrome (EPS) | 0 | Count | 165 | 389 | 3474 | 2 | 32 | 4062 |
|  |  | % within Epigastric pain syndrome (EPS) | 4.1% | 9.6% | 85.5% | 0.0% | 0.8% | 100.0% |
|  |  | % within Ethnicity | 61.8% | 78.1% | 74.0% | 100.0% | 74.4% | 73.8% |
|  |  | % of Total | 3.0% | 7.1% | 63.1% | 0.0% | 0.6% | 73.8% |
|  | 1 | Count | 102 | 109 | 1221 | 0 | 11 | 1443 |
|  |  | % within Epigastric pain syndrome (EPS) | 7.1% | 7.6% | 84.6% | 0.0% | 0.8% | 100.0% |
|  |  | % within Ethnicity | 38.2% | 21.9% | 26.0% | 0.0% | 25.6% | 26.2% |
|  |  | % of Total | 1.9% | 2.0% | 22.2% | 0.0% | 0.2% | 26.2% |
| Total | | Count | 267 | 498 | 4695 | 2 | 43 | 5505 |
|  |  | % within Epigastric pain syndrome (EPS) | 4.9% | 9.0% | 85.3% | 0.0% | 0.8% | 100.0% |
|  |  | % within Ethnicity | 100.0% | 100.0% | 100.0% | 100.0% | 100.0% | 100.0% |
|  |  | % of Total | 4.9% | 9.0% | 85.3% | 0.0% | 0.8% | 100.0% |

| **Chi-Square Tests** | | | |
| --- | --- | --- | --- |
|  | Value | df | Asymptotic Significance (2-sided) |
| Pearson Chi-Square | 25.483^a^ | 4 | .000 |
| Likelihood Ratio | 24.656 | 4 | .000 |
| N of Valid Cases | 5505 |  |  |
| a. 2 cells (20.0%) have expected count less than 5. The minimum expected count is .52. | | | |

| **Symmetric Measures^a^** | |
| --- | --- |
|  | Value |
| N of Valid Cases | 5505 |
| a. Correlation statistics are available for numeric data only. | |

**Epigastric pain syndrome (EPS) * ASA Grade**

| **Crosstab** | | | | | | | | |
| --- | --- | --- | --- | --- | --- | --- | --- | --- |
|  | | | ASA Grade | | | | | Total |
|  |  |  | ASA 1 | ASA 2 | ASA 3 | ASA 4 | ASA 5 |  |
| Epigastric pain syndrome (EPS) | 0 | Count | 2973 | 925 | 148 | 13 | 3 | 4062 |
|  |  | % within Epigastric pain syndrome (EPS) | 73.2% | 22.8% | 3.6% | 0.3% | 0.1% | 100.0% |
|  |  | % within ASA Grade | 77.7% | 66.0% | 59.9% | 48.1% | 50.0% | 73.8% |
|  |  | % of Total | 54.0% | 16.8% | 2.7% | 0.2% | 0.1% | 73.8% |
|  | 1 | Count | 851 | 476 | 99 | 14 | 3 | 1443 |
|  |  | % within Epigastric pain syndrome (EPS) | 59.0% | 33.0% | 6.9% | 1.0% | 0.2% | 100.0% |
|  |  | % within ASA Grade | 22.3% | 34.0% | 40.1% | 51.9% | 50.0% | 26.2% |
|  |  | % of Total | 15.5% | 8.6% | 1.8% | 0.3% | 0.1% | 26.2% |
| Total | | Count | 3824 | 1401 | 247 | 27 | 6 | 5505 |
|  |  | % within Epigastric pain syndrome (EPS) | 69.5% | 25.4% | 4.5% | 0.5% | 0.1% | 100.0% |
|  |  | % within ASA Grade | 100.0% | 100.0% | 100.0% | 100.0% | 100.0% | 100.0% |
|  |  | % of Total | 69.5% | 25.4% | 4.5% | 0.5% | 0.1% | 100.0% |

| **Chi-Square Tests** | | | |
| --- | --- | --- | --- |
|  | Value | df | Asymptotic Significance (2-sided) |
| Pearson Chi-Square | 110.126^a^ | 4 | .000 |
| Likelihood Ratio | 105.380 | 4 | .000 |
| N of Valid Cases | 5505 |  |  |
| a. 2 cells (20.0%) have expected count less than 5. The minimum expected count is 1.57. | | | |

| **Symmetric Measures^a^** | |
| --- | --- |
|  | Value |
| N of Valid Cases | 5505 |
| a. Correlation statistics are available for numeric data only. | |

**Epigastric pain syndrome (EPS) * Geographic**

| **Crosstab** | | | | | | |
| --- | --- | --- | --- | --- | --- | --- |
|  | | | Geographic | | | Total |
|  |  |  | Nomad life | Rural life | Urban life |  |
| Epigastric pain syndrome (EPS) | 0 | Count | 77 | 673 | 3312 | 4062 |
|  |  | % within Epigastric pain syndrome (EPS) | 1.9% | 16.6% | 81.5% | 100.0% |
|  |  | % within Geographic | 80.2% | 69.4% | 74.6% | 73.8% |
|  |  | % of Total | 1.4% | 12.2% | 60.2% | 73.8% |
|  | 1 | Count | 19 | 297 | 1127 | 1443 |
|  |  | % within Epigastric pain syndrome (EPS) | 1.3% | 20.6% | 78.1% | 100.0% |
|  |  | % within Geographic | 19.8% | 30.6% | 25.4% | 26.2% |
|  |  | % of Total | 0.3% | 5.4% | 20.5% | 26.2% |
| Total | | Count | 96 | 970 | 4439 | 5505 |
|  |  | % within Epigastric pain syndrome (EPS) | 1.7% | 17.6% | 80.6% | 100.0% |
|  |  | % within Geographic | 100.0% | 100.0% | 100.0% | 100.0% |
|  |  | % of Total | 1.7% | 17.6% | 80.6% | 100.0% |

| **Chi-Square Tests** | | | |
| --- | --- | --- | --- |
|  | Value | df | Asymptotic Significance (2-sided) |
| Pearson Chi-Square | 13.340^a^ | 2 | .001 |
| Likelihood Ratio | 13.158 | 2 | .001 |
| N of Valid Cases | 5505 |  |  |
| a. 0 cells (0.0%) have expected count less than 5. The minimum expected count is 25.16. | | | |

| **Symmetric Measures^a^** | |
| --- | --- |
|  | Value |
| N of Valid Cases | 5505 |
| a. Correlation statistics are available for numeric data only. | |

**Epigastric pain syndrome (EPS) * Hypertension requiring medication**

| **Crosstab** | | | | | |
| --- | --- | --- | --- | --- | --- |
|  | | | Hypertension requiring medication | | Total |
|  |  |  | 0 | 1 |  |
| Epigastric pain syndrome (EPS) | 0 | Count | 3700 | 362 | 4062 |
|  |  | % within Epigastric pain syndrome (EPS) | 91.1% | 8.9% | 100.0% |
|  |  | % within Hypertension requiring medication | 75.5% | 59.6% | 73.8% |
|  |  | % of Total | 67.2% | 6.6% | 73.8% |
|  | 1 | Count | 1198 | 245 | 1443 |
|  |  | % within Epigastric pain syndrome (EPS) | 83.0% | 17.0% | 100.0% |
|  |  | % within Hypertension requiring medication | 24.5% | 40.4% | 26.2% |
|  |  | % of Total | 21.8% | 4.5% | 26.2% |
| Total | | Count | 4898 | 607 | 5505 |
|  |  | % within Epigastric pain syndrome (EPS) | 89.0% | 11.0% | 100.0% |
|  |  | % within Hypertension requiring medication | 100.0% | 100.0% | 100.0% |
|  |  | % of Total | 89.0% | 11.0% | 100.0% |

| **Chi-Square Tests** | | | | | |
| --- | --- | --- | --- | --- | --- |
|  | Value | df | Asymptotic Significance (2-sided) | Exact Sig. (2-sided) | Exact Sig. (1-sided) |
| Pearson Chi-Square | 70.622^a^ | 1 | .000 |  |  |
| Continuity Correction^b^ | 69.803 | 1 | .000 |  |  |
| Likelihood Ratio | 65.276 | 1 | .000 |  |  |
| Fisher's Exact Test |  |  |  | .000 | .000 |
| Linear-by-Linear Association | 70.610 | 1 | .000 |  |  |
| N of Valid Cases | 5505 |  |  |  |  |
| a. 0 cells (0.0%) have expected count less than 5. The minimum expected count is 159.11. | | | | | |
| b. Computed only for a 2x2 table | | | | | |

| **Symmetric Measures** | | | | | |
| --- | --- | --- | --- | --- | --- |
|  | | Value | Asymptotic Standard Error^a^ | Approximate T^b^ | Approximate Significance |
| Interval by Interval | Pearson's R | .113 | .015 | 8.457 | .000^c^ |
| Ordinal by Ordinal | Spearman Correlation | .113 | .015 | 8.457 | .000^c^ |
| N of Valid Cases | | 5505 |  |  |  |
| a. Not assuming the null hypothesis. | | | | | |
| b. Using the asymptotic standard error assuming the null hypothesis. | | | | | |
| c. Based on normal approximation. | | | | | |

**Epigastric pain syndrome (EPS) * Diabetes Mellitus**

| **Crosstab** | | | | | |
| --- | --- | --- | --- | --- | --- |
|  | | | Diabetes Mellitus | | Total |
|  |  |  | 0 | 1 |  |
| Epigastric pain syndrome (EPS) | 0 | Count | 3820 | 242 | 4062 |
|  |  | % within Epigastric pain syndrome (EPS) | 94.0% | 6.0% | 100.0% |
|  |  | % within Diabetes Mellitus | 74.8% | 60.3% | 73.8% |
|  |  | % of Total | 69.4% | 4.4% | 73.8% |
|  | 1 | Count | 1284 | 159 | 1443 |
|  |  | % within Epigastric pain syndrome (EPS) | 89.0% | 11.0% | 100.0% |
|  |  | % within Diabetes Mellitus | 25.2% | 39.7% | 26.2% |
|  |  | % of Total | 23.3% | 2.9% | 26.2% |
| Total | | Count | 5104 | 401 | 5505 |
|  |  | % within Epigastric pain syndrome (EPS) | 92.7% | 7.3% | 100.0% |
|  |  | % within Diabetes Mellitus | 100.0% | 100.0% | 100.0% |
|  |  | % of Total | 92.7% | 7.3% | 100.0% |

| **Chi-Square Tests** | | | | | |
| --- | --- | --- | --- | --- | --- |
|  | Value | df | Asymptotic Significance (2-sided) | Exact Sig. (2-sided) | Exact Sig. (1-sided) |
| Pearson Chi-Square | 40.382^a^ | 1 | .000 |  |  |
| Continuity Correction^b^ | 39.636 | 1 | .000 |  |  |
| Likelihood Ratio | 37.275 | 1 | .000 |  |  |
| Fisher's Exact Test |  |  |  | .000 | .000 |
| Linear-by-Linear Association | 40.375 | 1 | .000 |  |  |
| N of Valid Cases | 5505 |  |  |  |  |
| a. 0 cells (0.0%) have expected count less than 5. The minimum expected count is 105.11. | | | | | |
| b. Computed only for a 2x2 table | | | | | |

| **Symmetric Measures** | | | | | |
| --- | --- | --- | --- | --- | --- |
|  | | Value | Asymptotic Standard Error^a^ | Approximate T^b^ | Approximate Significance |
| Interval by Interval | Pearson's R | .086 | .015 | 6.377 | .000^c^ |
| Ordinal by Ordinal | Spearman Correlation | .086 | .015 | 6.377 | .000^c^ |
| N of Valid Cases | | 5505 |  |  |  |
| a. Not assuming the null hypothesis. | | | | | |
| b. Using the asymptotic standard error assuming the null hypothesis. | | | | | |
| c. Based on normal approximation. | | | | | |

**Epigastric pain syndrome (EPS) * Autoimmune diseases**

| **Crosstab** | | | | | |
| --- | --- | --- | --- | --- | --- |
|  | | | Autoimmune diseases | | Total |
|  |  |  | 0 | 1 |  |
| Epigastric pain syndrome (EPS) | 0 | Count | 3968 | 94 | 4062 |
|  |  | % within Epigastric pain syndrome (EPS) | 97.7% | 2.3% | 100.0% |
|  |  | % within Autoimmune diseases | 74.0% | 65.7% | 73.8% |
|  |  | % of Total | 72.1% | 1.7% | 73.8% |
|  | 1 | Count | 1394 | 49 | 1443 |
|  |  | % within Epigastric pain syndrome (EPS) | 96.6% | 3.4% | 100.0% |
|  |  | % within Autoimmune diseases | 26.0% | 34.3% | 26.2% |
|  |  | % of Total | 25.3% | 0.9% | 26.2% |
| Total | | Count | 5362 | 143 | 5505 |
|  |  | % within Epigastric pain syndrome (EPS) | 97.4% | 2.6% | 100.0% |
|  |  | % within Autoimmune diseases | 100.0% | 100.0% | 100.0% |
|  |  | % of Total | 97.4% | 2.6% | 100.0% |

| **Chi-Square Tests** | | | | | |
| --- | --- | --- | --- | --- | --- |
|  | Value | df | Asymptotic Significance (2-sided) | Exact Sig. (2-sided) | Exact Sig. (1-sided) |
| Pearson Chi-Square | 4.923^a^ | 1 | .027 |  |  |
| Continuity Correction^b^ | 4.505 | 1 | .034 |  |  |
| Likelihood Ratio | 4.656 | 1 | .031 |  |  |
| Fisher's Exact Test |  |  |  | .034 | .019 |
| Linear-by-Linear Association | 4.922 | 1 | .027 |  |  |
| N of Valid Cases | 5505 |  |  |  |  |
| a. 0 cells (0.0%) have expected count less than 5. The minimum expected count is 37.48. | | | | | |
| b. Computed only for a 2x2 table | | | | | |

| **Symmetric Measures** | | | | | |
| --- | --- | --- | --- | --- | --- |
|  | | Value | Asymptotic Standard Error^a^ | Approximate T^b^ | Approximate Significance |
| Interval by Interval | Pearson's R | .030 | .015 | 2.219 | .027^c^ |
| Ordinal by Ordinal | Spearman Correlation | .030 | .015 | 2.219 | .027^c^ |
| N of Valid Cases | | 5505 |  |  |  |
| a. Not assuming the null hypothesis. | | | | | |
| b. Using the asymptotic standard error assuming the null hypothesis. | | | | | |
| c. Based on normal approximation. | | | | | |

**Epigastric pain syndrome (EPS) * Headache or migraine**

| **Crosstab** | | | | | |
| --- | --- | --- | --- | --- | --- |
|  | | | Headache or migraine | | Total |
|  |  |  | 0 | 1 |  |
| Epigastric pain syndrome (EPS) | 0 | Count | 3704 | 358 | 4062 |
|  |  | % within Epigastric pain syndrome (EPS) | 91.2% | 8.8% | 100.0% |
|  |  | % within Headache or migraine | 74.9% | 63.6% | 73.8% |
|  |  | % of Total | 67.3% | 6.5% | 73.8% |
|  | 1 | Count | 1238 | 205 | 1443 |
|  |  | % within Epigastric pain syndrome (EPS) | 85.8% | 14.2% | 100.0% |
|  |  | % within Headache or migraine | 25.1% | 36.4% | 26.2% |
|  |  | % of Total | 22.5% | 3.7% | 26.2% |
| Total | | Count | 4942 | 563 | 5505 |
|  |  | % within Epigastric pain syndrome (EPS) | 89.8% | 10.2% | 100.0% |
|  |  | % within Headache or migraine | 100.0% | 100.0% | 100.0% |
|  |  | % of Total | 89.8% | 10.2% | 100.0% |

| **Chi-Square Tests** | | | | | |
| --- | --- | --- | --- | --- | --- |
|  | Value | df | Asymptotic Significance (2-sided) | Exact Sig. (2-sided) | Exact Sig. (1-sided) |
| Pearson Chi-Square | 33.731^a^ | 1 | .000 |  |  |
| Continuity Correction^b^ | 33.146 | 1 | .000 |  |  |
| Likelihood Ratio | 31.719 | 1 | .000 |  |  |
| Fisher's Exact Test |  |  |  | .000 | .000 |
| Linear-by-Linear Association | 33.725 | 1 | .000 |  |  |
| N of Valid Cases | 5505 |  |  |  |  |
| a. 0 cells (0.0%) have expected count less than 5. The minimum expected count is 147.58. | | | | | |
| b. Computed only for a 2x2 table | | | | | |

| **Symmetric Measures** | | | | | |
| --- | --- | --- | --- | --- | --- |
|  | | Value | Asymptotic Standard Error^a^ | Approximate T^b^ | Approximate Significance |
| Interval by Interval | Pearson's R | .078 | .015 | 5.825 | .000^c^ |
| Ordinal by Ordinal | Spearman Correlation | .078 | .015 | 5.825 | .000^c^ |
| N of Valid Cases | | 5505 |  |  |  |
| a. Not assuming the null hypothesis. | | | | | |
| b. Using the asymptotic standard error assuming the null hypothesis. | | | | | |
| c. Based on normal approximation. | | | | | |

**Epigastric pain syndrome (EPS) * Chronic immunosuppression**

| **Crosstab** | | | | | |
| --- | --- | --- | --- | --- | --- |
|  | | | Chronic immunosuppression | | Total |
|  |  |  | 0 | 1 |  |
| Epigastric pain syndrome (EPS) | 0 | Count | 4049 | 13 | 4062 |
|  |  | % within Epigastric pain syndrome (EPS) | 99.7% | 0.3% | 100.0% |
|  |  | % within Chronic immunosuppression | 73.9% | 56.5% | 73.8% |
|  |  | % of Total | 73.6% | 0.2% | 73.8% |
|  | 1 | Count | 1433 | 10 | 1443 |
|  |  | % within Epigastric pain syndrome (EPS) | 99.3% | 0.7% | 100.0% |
|  |  | % within Chronic immunosuppression | 26.1% | 43.5% | 26.2% |
|  |  | % of Total | 26.0% | 0.2% | 26.2% |
| Total | | Count | 5482 | 23 | 5505 |
|  |  | % within Epigastric pain syndrome (EPS) | 99.6% | 0.4% | 100.0% |
|  |  | % within Chronic immunosuppression | 100.0% | 100.0% | 100.0% |
|  |  | % of Total | 99.6% | 0.4% | 100.0% |

| **Chi-Square Tests** | | | | | |
| --- | --- | --- | --- | --- | --- |
|  | Value | df | Asymptotic Significance (2-sided) | Exact Sig. (2-sided) | Exact Sig. (1-sided) |
| Pearson Chi-Square | 3.560^a^ | 1 | .059 |  |  |
| Continuity Correction^b^ | 2.720 | 1 | .099 |  |  |
| Likelihood Ratio | 3.205 | 1 | .073 |  |  |
| Fisher's Exact Test |  |  |  | .092 | .054 |
| Linear-by-Linear Association | 3.559 | 1 | .059 |  |  |
| N of Valid Cases | 5505 |  |  |  |  |
| a. 0 cells (0.0%) have expected count less than 5. The minimum expected count is 6.03. | | | | | |
| b. Computed only for a 2x2 table | | | | | |

| **Symmetric Measures** | | | | | |
| --- | --- | --- | --- | --- | --- |
|  | | Value | Asymptotic Standard Error^a^ | Approximate T^b^ | Approximate Significance |
| Interval by Interval | Pearson's R | .025 | .015 | 1.887 | .059^c^ |
| Ordinal by Ordinal | Spearman Correlation | .025 | .015 | 1.887 | .059^c^ |
| N of Valid Cases | | 5505 |  |  |  |
| a. Not assuming the null hypothesis. | | | | | |
| b. Using the asymptotic standard error assuming the null hypothesis. | | | | | |
| c. Based on normal approximation. | | | | | |

**Epigastric pain syndrome (EPS) * Anemia**

| **Crosstab** | | | | | |
| --- | --- | --- | --- | --- | --- |
|  | | | Anemia | | Total |
|  |  |  | 0 | 1 |  |
| Epigastric pain syndrome (EPS) | 0 | Count | 3580 | 482 | 4062 |
|  |  | % within Epigastric pain syndrome (EPS) | 88.1% | 11.9% | 100.0% |
|  |  | % within Anemia | 74.8% | 67.2% | 73.8% |
|  |  | % of Total | 65.0% | 8.8% | 73.8% |
|  | 1 | Count | 1208 | 235 | 1443 |
|  |  | % within Epigastric pain syndrome (EPS) | 83.7% | 16.3% | 100.0% |
|  |  | % within Anemia | 25.2% | 32.8% | 26.2% |
|  |  | % of Total | 21.9% | 4.3% | 26.2% |
| Total | | Count | 4788 | 717 | 5505 |
|  |  | % within Epigastric pain syndrome (EPS) | 87.0% | 13.0% | 100.0% |
|  |  | % within Anemia | 100.0% | 100.0% | 100.0% |
|  |  | % of Total | 87.0% | 13.0% | 100.0% |

| **Chi-Square Tests** | | | | | |
| --- | --- | --- | --- | --- | --- |
|  | Value | df | Asymptotic Significance (2-sided) | Exact Sig. (2-sided) | Exact Sig. (1-sided) |
| Pearson Chi-Square | 18.358^a^ | 1 | .000 |  |  |
| Continuity Correction^b^ | 17.970 | 1 | .000 |  |  |
| Likelihood Ratio | 17.632 | 1 | .000 |  |  |
| Fisher's Exact Test |  |  |  | .000 | .000 |
| Linear-by-Linear Association | 18.355 | 1 | .000 |  |  |
| N of Valid Cases | 5505 |  |  |  |  |
| a. 0 cells (0.0%) have expected count less than 5. The minimum expected count is 187.94. | | | | | |
| b. Computed only for a 2x2 table | | | | | |

| **Symmetric Measures** | | | | | |
| --- | --- | --- | --- | --- | --- |
|  | | Value | Asymptotic Standard Error^a^ | Approximate T^b^ | Approximate Significance |
| Interval by Interval | Pearson's R | .058 | .014 | 4.291 | .000^c^ |
| Ordinal by Ordinal | Spearman Correlation | .058 | .014 | 4.291 | .000^c^ |
| N of Valid Cases | | 5505 |  |  |  |
| a. Not assuming the null hypothesis. | | | | | |
| b. Using the asymptotic standard error assuming the null hypothesis. | | | | | |
| c. Based on normal approximation. | | | | | |

**Epigastric pain syndrome (EPS) * Patients allergic to certain substances**

| **Crosstab** | | | | | |
| --- | --- | --- | --- | --- | --- |
|  | | | Patients allergic to certain substances | | Total |
|  |  |  | 0 | 1 |  |
| Epigastric pain syndrome (EPS) | 0 | Count | 3663 | 399 | 4062 |
|  |  | % within Epigastric pain syndrome (EPS) | 90.2% | 9.8% | 100.0% |
|  |  | % within Patients allergic to certain substances | 74.8% | 65.7% | 73.8% |
|  |  | % of Total | 66.5% | 7.2% | 73.8% |
|  | 1 | Count | 1235 | 208 | 1443 |
|  |  | % within Epigastric pain syndrome (EPS) | 85.6% | 14.4% | 100.0% |
|  |  | % within Patients allergic to certain substances | 25.2% | 34.3% | 26.2% |
|  |  | % of Total | 22.4% | 3.8% | 26.2% |
| Total | | Count | 4898 | 607 | 5505 |
|  |  | % within Epigastric pain syndrome (EPS) | 89.0% | 11.0% | 100.0% |
|  |  | % within Patients allergic to certain substances | 100.0% | 100.0% | 100.0% |
|  |  | % of Total | 89.0% | 11.0% | 100.0% |

| **Chi-Square Tests** | | | | | |
| --- | --- | --- | --- | --- | --- |
|  | Value | df | Asymptotic Significance (2-sided) | Exact Sig. (2-sided) | Exact Sig. (1-sided) |
| Pearson Chi-Square | 22.882^a^ | 1 | .000 |  |  |
| Continuity Correction^b^ | 22.417 | 1 | .000 |  |  |
| Likelihood Ratio | 21.770 | 1 | .000 |  |  |
| Fisher's Exact Test |  |  |  | .000 | .000 |
| Linear-by-Linear Association | 22.878 | 1 | .000 |  |  |
| N of Valid Cases | 5505 |  |  |  |  |
| a. 0 cells (0.0%) have expected count less than 5. The minimum expected count is 159.11. | | | | | |
| b. Computed only for a 2x2 table | | | | | |

| **Symmetric Measures** | | | | | |
| --- | --- | --- | --- | --- | --- |
|  | | Value | Asymptotic Standard Error^a^ | Approximate T^b^ | Approximate Significance |
| Interval by Interval | Pearson's R | .064 | .014 | 4.793 | .000^c^ |
| Ordinal by Ordinal | Spearman Correlation | .064 | .014 | 4.793 | .000^c^ |
| N of Valid Cases | | 5505 |  |  |  |
| a. Not assuming the null hypothesis. | | | | | |
| b. Using the asymptotic standard error assuming the null hypothesis. | | | | | |
| c. Based on normal approximation. | | | | | |

**Epigastric pain syndrome (EPS) * Chronic obstructive pulmonary disease (COPD)**

| **Crosstab** | | | | | |
| --- | --- | --- | --- | --- | --- |
|  | | | Chronic obstructive pulmonary disease (COPD) | | Total |
|  |  |  | 0 | 1 |  |
| Epigastric pain syndrome (EPS) | 0 | Count | 4032 | 30 | 4062 |
|  |  | % within Epigastric pain syndrome (EPS) | 99.3% | 0.7% | 100.0% |
|  |  | % within Chronic obstructive pulmonary disease (COPD) | 73.9% | 60.0% | 73.8% |
|  |  | % of Total | 73.2% | 0.5% | 73.8% |
|  | 1 | Count | 1423 | 20 | 1443 |
|  |  | % within Epigastric pain syndrome (EPS) | 98.6% | 1.4% | 100.0% |
|  |  | % within Chronic obstructive pulmonary disease (COPD) | 26.1% | 40.0% | 26.2% |
|  |  | % of Total | 25.8% | 0.4% | 26.2% |
| Total | | Count | 5455 | 50 | 5505 |
|  |  | % within Epigastric pain syndrome (EPS) | 99.1% | 0.9% | 100.0% |
|  |  | % within Chronic obstructive pulmonary disease (COPD) | 100.0% | 100.0% | 100.0% |
|  |  | % of Total | 99.1% | 0.9% | 100.0% |

| **Chi-Square Tests** | | | | | |
| --- | --- | --- | --- | --- | --- |
|  | Value | df | Asymptotic Significance (2-sided) | Exact Sig. (2-sided) | Exact Sig. (1-sided) |
| Pearson Chi-Square | 4.959^a^ | 1 | .026 |  |  |
| Continuity Correction^b^ | 4.266 | 1 | .039 |  |  |
| Likelihood Ratio | 4.540 | 1 | .033 |  |  |
| Fisher's Exact Test |  |  |  | .035 | .023 |
| Linear-by-Linear Association | 4.958 | 1 | .026 |  |  |
| N of Valid Cases | 5505 |  |  |  |  |
| a. 0 cells (0.0%) have expected count less than 5. The minimum expected count is 13.11. | | | | | |
| b. Computed only for a 2x2 table | | | | | |

| **Symmetric Measures** | | | | | |
| --- | --- | --- | --- | --- | --- |
|  | | Value | Asymptotic Standard Error^a^ | Approximate T^b^ | Approximate Significance |
| Interval by Interval | Pearson's R | .030 | .015 | 2.228 | .026^c^ |
| Ordinal by Ordinal | Spearman Correlation | .030 | .015 | 2.228 | .026^c^ |
| N of Valid Cases | | 5505 |  |  |  |
| a. Not assuming the null hypothesis. | | | | | |
| b. Using the asymptotic standard error assuming the null hypothesis. | | | | | |
| c. Based on normal approximation. | | | | | |

**Epigastric pain syndrome (EPS) * COVID-19 infection**

| **Crosstab** | | | | | |
| --- | --- | --- | --- | --- | --- |
|  | | | COVID-19 infection | | Total |
|  |  |  | 0 | 1 |  |
| Epigastric pain syndrome (EPS) | 0 | Count | 2320 | 1742 | 4062 |
|  |  | % within Epigastric pain syndrome (EPS) | 57.1% | 42.9% | 100.0% |
|  |  | % within COVID-19 infection | 75.6% | 71.5% | 73.8% |
|  |  | % of Total | 42.1% | 31.6% | 73.8% |
|  | 1 | Count | 750 | 693 | 1443 |
|  |  | % within Epigastric pain syndrome (EPS) | 52.0% | 48.0% | 100.0% |
|  |  | % within COVID-19 infection | 24.4% | 28.5% | 26.2% |
|  |  | % of Total | 13.6% | 12.6% | 26.2% |
| Total | | Count | 3070 | 2435 | 5505 |
|  |  | % within Epigastric pain syndrome (EPS) | 55.8% | 44.2% | 100.0% |
|  |  | % within COVID-19 infection | 100.0% | 100.0% | 100.0% |
|  |  | % of Total | 55.8% | 44.2% | 100.0% |

| **Chi-Square Tests** | | | | | |
| --- | --- | --- | --- | --- | --- |
|  | Value | df | Asymptotic Significance (2-sided) | Exact Sig. (2-sided) | Exact Sig. (1-sided) |
| Pearson Chi-Square | 11.402^a^ | 1 | .001 |  |  |
| Continuity Correction^b^ | 11.195 | 1 | .001 |  |  |
| Likelihood Ratio | 11.368 | 1 | .001 |  |  |
| Fisher's Exact Test |  |  |  | .001 | .000 |
| Linear-by-Linear Association | 11.400 | 1 | .001 |  |  |
| N of Valid Cases | 5505 |  |  |  |  |
| a. 0 cells (0.0%) have expected count less than 5. The minimum expected count is 638.28. | | | | | |
| b. Computed only for a 2x2 table | | | | | |

| **Symmetric Measures** | | | | | |
| --- | --- | --- | --- | --- | --- |
|  | | Value | Asymptotic Standard Error^a^ | Approximate T^b^ | Approximate Significance |
| Interval by Interval | Pearson's R | .046 | .014 | 3.380 | .001^c^ |
| Ordinal by Ordinal | Spearman Correlation | .046 | .014 | 3.380 | .001^c^ |
| N of Valid Cases | | 5505 |  |  |  |
| a. Not assuming the null hypothesis. | | | | | |
| b. Using the asymptotic standard error assuming the null hypothesis. | | | | | |
| c. Based on normal approximation. | | | | | |

**Epigastric pain syndrome (EPS) * Open abdominal surgery/laparotomy**

| **Crosstab** | | | | | |
| --- | --- | --- | --- | --- | --- |
|  | | | Open abdominal surgery/laparotomy | | Total |
|  |  |  | 0 | 1 |  |
| Epigastric pain syndrome (EPS) | 0 | Count | 3352 | 710 | 4062 |
|  |  | % within Epigastric pain syndrome (EPS) | 82.5% | 17.5% | 100.0% |
|  |  | % within Open abdominal surgery/laparotomy | 76.5% | 63.2% | 73.8% |
|  |  | % of Total | 60.9% | 12.9% | 73.8% |
|  | 1 | Count | 1030 | 413 | 1443 |
|  |  | % within Epigastric pain syndrome (EPS) | 71.4% | 28.6% | 100.0% |
|  |  | % within Open abdominal surgery/laparotomy | 23.5% | 36.8% | 26.2% |
|  |  | % of Total | 18.7% | 7.5% | 26.2% |
| Total | | Count | 4382 | 1123 | 5505 |
|  |  | % within Epigastric pain syndrome (EPS) | 79.6% | 20.4% | 100.0% |
|  |  | % within Open abdominal surgery/laparotomy | 100.0% | 100.0% | 100.0% |
|  |  | % of Total | 79.6% | 20.4% | 100.0% |

| **Chi-Square Tests** | | | | | |
| --- | --- | --- | --- | --- | --- |
|  | Value | df | Asymptotic Significance (2-sided) | Exact Sig. (2-sided) | Exact Sig. (1-sided) |
| Pearson Chi-Square | 81.400^a^ | 1 | .000 |  |  |
| Continuity Correction^b^ | 80.716 | 1 | .000 |  |  |
| Likelihood Ratio | 77.291 | 1 | .000 |  |  |
| Fisher's Exact Test |  |  |  | .000 | .000 |
| Linear-by-Linear Association | 81.386 | 1 | .000 |  |  |
| N of Valid Cases | 5505 |  |  |  |  |
| a. 0 cells (0.0%) have expected count less than 5. The minimum expected count is 294.37. | | | | | |
| b. Computed only for a 2x2 table | | | | | |

| **Symmetric Measures** | | | | | |
| --- | --- | --- | --- | --- | --- |
|  | | Value | Asymptotic Standard Error^a^ | Approximate T^b^ | Approximate Significance |
| Interval by Interval | Pearson's R | .122 | .014 | 9.088 | .000^c^ |
| Ordinal by Ordinal | Spearman Correlation | .122 | .014 | 9.088 | .000^c^ |
| N of Valid Cases | | 5505 |  |  |  |
| a. Not assuming the null hypothesis. | | | | | |
| b. Using the asymptotic standard error assuming the null hypothesis. | | | | | |
| c. Based on normal approximation. | | | | | |

**Epigastric pain syndrome (EPS) * (CFQ) Scale**

| **Crosstab** | | | | | | |
| --- | --- | --- | --- | --- | --- | --- |
|  | | | (CFQ) Scale | | | Total |
|  |  |  | Low fatigue | Moderate Fatigue | Severe Fatigue |  |
| Epigastric pain syndrome (EPS) | 0 | Count | 1233 | 2522 | 307 | 4062 |
|  |  | % within Epigastric pain syndrome (EPS) | 30.4% | 62.1% | 7.6% | 100.0% |
|  |  | % within (CFQ) Scale | 82.9% | 72.5% | 57.2% | 73.8% |
|  |  | % of Total | 22.4% | 45.8% | 5.6% | 73.8% |
|  | 1 | Count | 255 | 958 | 230 | 1443 |
|  |  | % within Epigastric pain syndrome (EPS) | 17.7% | 66.4% | 15.9% | 100.0% |
|  |  | % within (CFQ) Scale | 17.1% | 27.5% | 42.8% | 26.2% |
|  |  | % of Total | 4.6% | 17.4% | 4.2% | 26.2% |
| Total | | Count | 1488 | 3480 | 537 | 5505 |
|  |  | % within Epigastric pain syndrome (EPS) | 27.0% | 63.2% | 9.8% | 100.0% |
|  |  | % within (CFQ) Scale | 100.0% | 100.0% | 100.0% | 100.0% |
|  |  | % of Total | 27.0% | 63.2% | 9.8% | 100.0% |

| **Chi-Square Tests** | | | |
| --- | --- | --- | --- |
|  | Value | df | Asymptotic Significance (2-sided) |
| Pearson Chi-Square | 143.154^a^ | 2 | .000 |
| Likelihood Ratio | 141.583 | 2 | .000 |
| N of Valid Cases | 5505 |  |  |
| a. 0 cells (0.0%) have expected count less than 5. The minimum expected count is 140.76. | | | |

| **Symmetric Measures^a^** | |
| --- | --- |
|  | Value |
| N of Valid Cases | 5505 |
| a. Correlation statistics are available for numeric data only. | |

**Epigastric pain syndrome (EPS) * Smoking**

| **Crosstab** | | | | | | | | |
| --- | --- | --- | --- | --- | --- | --- | --- | --- |
|  | | | Smoking | | | | | Total |
|  |  |  |  | A daily smoker | An occasional smoker | Ex-smoker | Non-smoker |  |
| Epigastric pain syndrome (EPS) | 0 | Count | 0 | 630 | 470 | 197 | 2765 | 4062 |
|  |  | % within Epigastric pain syndrome (EPS) | 0.0% | 15.5% | 11.6% | 4.8% | 68.1% | 100.0% |
|  |  | % within Smoking | 0.0% | 67.7% | 75.0% | 71.6% | 75.3% | 73.8% |
|  |  | % of Total | 0.0% | 11.4% | 8.5% | 3.6% | 50.2% | 73.8% |
|  | 1 | Count | 1 | 300 | 157 | 78 | 907 | 1443 |
|  |  | % within Epigastric pain syndrome (EPS) | 0.1% | 20.8% | 10.9% | 5.4% | 62.9% | 100.0% |
|  |  | % within Smoking | 100.0% | 32.3% | 25.0% | 28.4% | 24.7% | 26.2% |
|  |  | % of Total | 0.0% | 5.4% | 2.9% | 1.4% | 16.5% | 26.2% |
| Total | | Count | 1 | 930 | 627 | 275 | 3672 | 5505 |
|  |  | % within Epigastric pain syndrome (EPS) | 0.0% | 16.9% | 11.4% | 5.0% | 66.7% | 100.0% |
|  |  | % within Smoking | 100.0% | 100.0% | 100.0% | 100.0% | 100.0% | 100.0% |
|  |  | % of Total | 0.0% | 16.9% | 11.4% | 5.0% | 66.7% | 100.0% |

| **Chi-Square Tests** | | | |
| --- | --- | --- | --- |
|  | Value | df | Asymptotic Significance (2-sided) |
| Pearson Chi-Square | 25.833^a^ | 4 | .000 |
| Likelihood Ratio | 24.981 | 4 | .000 |
| N of Valid Cases | 5505 |  |  |
| a. 2 cells (20.0%) have expected count less than 5. The minimum expected count is .26. | | | |

| **Symmetric Measures^a^** | |
| --- | --- |
|  | Value |
| N of Valid Cases | 5505 |
| a. Correlation statistics are available for numeric data only. | |

**Epigastric pain syndrome (EPS) * Alcohol**

| **Crosstab** | | | | | | | | |
| --- | --- | --- | --- | --- | --- | --- | --- | --- |
|  | | | Alcohol | | | | | Total |
|  |  |  |  | Binge Drinking | Drinking alcohol in moderation | Heavy Alcohol Use | Not drinking alcohol |  |
| Epigastric pain syndrome (EPS) | 0 | Count | 0 | 6 | 164 | 5 | 3887 | 4062 |
|  |  | % within Epigastric pain syndrome (EPS) | 0.0% | 0.1% | 4.0% | 0.1% | 95.7% | 100.0% |
|  |  | % within Alcohol | 0.0% | 66.7% | 67.5% | 71.4% | 74.1% | 73.8% |
|  |  | % of Total | 0.0% | 0.1% | 3.0% | 0.1% | 70.6% | 73.8% |
|  | 1 | Count | 1 | 3 | 79 | 2 | 1358 | 1443 |
|  |  | % within Epigastric pain syndrome (EPS) | 0.1% | 0.2% | 5.5% | 0.1% | 94.1% | 100.0% |
|  |  | % within Alcohol | 100.0% | 33.3% | 32.5% | 28.6% | 25.9% | 26.2% |
|  |  | % of Total | 0.0% | 0.1% | 1.4% | 0.0% | 24.7% | 26.2% |
| Total | | Count | 1 | 9 | 243 | 7 | 5245 | 5505 |
|  |  | % within Epigastric pain syndrome (EPS) | 0.0% | 0.2% | 4.4% | 0.1% | 95.3% | 100.0% |
|  |  | % within Alcohol | 100.0% | 100.0% | 100.0% | 100.0% | 100.0% | 100.0% |
|  |  | % of Total | 0.0% | 0.2% | 4.4% | 0.1% | 95.3% | 100.0% |

| **Chi-Square Tests** | | | |
| --- | --- | --- | --- |
|  | Value | df | Asymptotic Significance (2-sided) |
| Pearson Chi-Square | 8.334^a^ | 4 | .080 |
| Likelihood Ratio | 7.961 | 4 | .093 |
| N of Valid Cases | 5505 |  |  |
| a. 4 cells (40.0%) have expected count less than 5. The minimum expected count is .26. | | | |

| **Symmetric Measures^a^** | |
| --- | --- |
|  | Value |
| N of Valid Cases | 5505 |
| a. Correlation statistics are available for numeric data only. | |

**Epigastric pain syndrome (EPS) * PSS Scale**

| **Crosstab** | | | | | | |
| --- | --- | --- | --- | --- | --- | --- |
|  | | | PSS Scale | | | Total |
|  |  |  | High perceived stress | Low stress | Moderate stress |  |
| Epigastric pain syndrome (EPS) | 0 | Count | 524 | 394 | 3144 | 4062 |
|  |  | % within Epigastric pain syndrome (EPS) | 12.9% | 9.7% | 77.4% | 100.0% |
|  |  | % within PSS Scale | 63.9% | 84.0% | 74.6% | 73.8% |
|  |  | % of Total | 9.5% | 7.2% | 57.1% | 73.8% |
|  | 1 | Count | 296 | 75 | 1072 | 1443 |
|  |  | % within Epigastric pain syndrome (EPS) | 20.5% | 5.2% | 74.3% | 100.0% |
|  |  | % within PSS Scale | 36.1% | 16.0% | 25.4% | 26.2% |
|  |  | % of Total | 5.4% | 1.4% | 19.5% | 26.2% |
| Total | | Count | 820 | 469 | 4216 | 5505 |
|  |  | % within Epigastric pain syndrome (EPS) | 14.9% | 8.5% | 76.6% | 100.0% |
|  |  | % within PSS Scale | 100.0% | 100.0% | 100.0% | 100.0% |
|  |  | % of Total | 14.9% | 8.5% | 76.6% | 100.0% |

| **Chi-Square Tests** | | | |
| --- | --- | --- | --- |
|  | Value | df | Asymptotic Significance (2-sided) |
| Pearson Chi-Square | 68.104^a^ | 2 | .000 |
| Likelihood Ratio | 68.151 | 2 | .000 |
| N of Valid Cases | 5505 |  |  |
| a. 0 cells (0.0%) have expected count less than 5. The minimum expected count is 122.94. | | | |

| **Symmetric Measures^a^** | |
| --- | --- |
|  | Value |
| N of Valid Cases | 5505 |
| a. Correlation statistics are available for numeric data only. | |

**Postprandial distress syndrome (PPDS) * Sex**

| **Crosstab** | | | | | |
| --- | --- | --- | --- | --- | --- |
|  | | | Sex | | Total |
|  |  |  | Female | Male |  |
| Postprandial distress syndrome (PPDS) | 0 | Count | 2316 | 1861 | 4177 |
|  |  | % within Postprandial distress syndrome (PPDS) | 55.4% | 44.6% | 100.0% |
|  |  | % within Sex | 72.3% | 80.9% | 75.9% |
|  |  | % of Total | 42.1% | 33.8% | 75.9% |
|  | 1 | Count | 888 | 440 | 1328 |
|  |  | % within Postprandial distress syndrome (PPDS) | 66.9% | 33.1% | 100.0% |
|  |  | % within Sex | 27.7% | 19.1% | 24.1% |
|  |  | % of Total | 16.1% | 8.0% | 24.1% |
| Total | | Count | 3204 | 2301 | 5505 |
|  |  | % within Postprandial distress syndrome (PPDS) | 58.2% | 41.8% | 100.0% |
|  |  | % within Sex | 100.0% | 100.0% | 100.0% |
|  |  | % of Total | 58.2% | 41.8% | 100.0% |

| **Chi-Square Tests** | | | | | |
| --- | --- | --- | --- | --- | --- |
|  | Value | df | Asymptotic Significance (2-sided) | Exact Sig. (2-sided) | Exact Sig. (1-sided) |
| Pearson Chi-Square | 54.028^a^ | 1 | .000 |  |  |
| Continuity Correction^b^ | 53.559 | 1 | .000 |  |  |
| Likelihood Ratio | 55.007 | 1 | .000 |  |  |
| Fisher's Exact Test |  |  |  | .000 | .000 |
| N of Valid Cases | 5505 |  |  |  |  |
| a. 0 cells (0.0%) have expected count less than 5. The minimum expected count is 555.08. | | | | | |
| b. Computed only for a 2x2 table | | | | | |

| **Symmetric Measures^a^** | |
| --- | --- |
|  | Value |
| N of Valid Cases | 5505 |
| a. Correlation statistics are available for numeric data only. | |

**Postprandial distress syndrome (PPDS) * Ethnicity**

| **Crosstab** | | | | | | | | |
| --- | --- | --- | --- | --- | --- | --- | --- | --- |
|  | | | Ethnicity | | | | | Total |
|  |  |  | Afro-Caribbean | Asian | Caucasian | Hispanic | Other |  |
| Postprandial distress syndrome (PPDS) | 0 | Count | 179 | 391 | 3575 | 2 | 30 | 4177 |
|  |  | % within Postprandial distress syndrome (PPDS) | 4.3% | 9.4% | 85.6% | 0.0% | 0.7% | 100.0% |
|  |  | % within Ethnicity | 67.0% | 78.5% | 76.1% | 100.0% | 69.8% | 75.9% |
|  |  | % of Total | 3.3% | 7.1% | 64.9% | 0.0% | 0.5% | 75.9% |
|  | 1 | Count | 88 | 107 | 1120 | 0 | 13 | 1328 |
|  |  | % within Postprandial distress syndrome (PPDS) | 6.6% | 8.1% | 84.3% | 0.0% | 1.0% | 100.0% |
|  |  | % within Ethnicity | 33.0% | 21.5% | 23.9% | 0.0% | 30.2% | 24.1% |
|  |  | % of Total | 1.6% | 1.9% | 20.3% | 0.0% | 0.2% | 24.1% |
| Total | | Count | 267 | 498 | 4695 | 2 | 43 | 5505 |
|  |  | % within Postprandial distress syndrome (PPDS) | 4.9% | 9.0% | 85.3% | 0.0% | 0.8% | 100.0% |
|  |  | % within Ethnicity | 100.0% | 100.0% | 100.0% | 100.0% | 100.0% | 100.0% |
|  |  | % of Total | 4.9% | 9.0% | 85.3% | 0.0% | 0.8% | 100.0% |

| **Chi-Square Tests** | | | |
| --- | --- | --- | --- |
|  | Value | df | Asymptotic Significance (2-sided) |
| Pearson Chi-Square | 14.977^a^ | 4 | .005 |
| Likelihood Ratio | 14.669 | 4 | .005 |
| N of Valid Cases | 5505 |  |  |
| a. 2 cells (20.0%) have expected count less than 5. The minimum expected count is .48. | | | |

| **Symmetric Measures^a^** | |
| --- | --- |
|  | Value |
| N of Valid Cases | 5505 |
| a. Correlation statistics are available for numeric data only. | |

**Postprandial distress syndrome (PPDS) * ASA Grade**

| **Crosstab** | | | | | | | | |
| --- | --- | --- | --- | --- | --- | --- | --- | --- |
|  | | | ASA Grade | | | | | Total |
|  |  |  | ASA 1 | ASA 2 | ASA 3 | ASA 4 | ASA 5 |  |
| Postprandial distress syndrome (PPDS) | 0 | Count | 2994 | 997 | 169 | 12 | 5 | 4177 |
|  |  | % within Postprandial distress syndrome (PPDS) | 71.7% | 23.9% | 4.0% | 0.3% | 0.1% | 100.0% |
|  |  | % within ASA Grade | 78.3% | 71.2% | 68.4% | 44.4% | 83.3% | 75.9% |
|  |  | % of Total | 54.4% | 18.1% | 3.1% | 0.2% | 0.1% | 75.9% |
|  | 1 | Count | 830 | 404 | 78 | 15 | 1 | 1328 |
|  |  | % within Postprandial distress syndrome (PPDS) | 62.5% | 30.4% | 5.9% | 1.1% | 0.1% | 100.0% |
|  |  | % within ASA Grade | 21.7% | 28.8% | 31.6% | 55.6% | 16.7% | 24.1% |
|  |  | % of Total | 15.1% | 7.3% | 1.4% | 0.3% | 0.0% | 24.1% |
| Total | | Count | 3824 | 1401 | 247 | 27 | 6 | 5505 |
|  |  | % within Postprandial distress syndrome (PPDS) | 69.5% | 25.4% | 4.5% | 0.5% | 0.1% | 100.0% |
|  |  | % within ASA Grade | 100.0% | 100.0% | 100.0% | 100.0% | 100.0% | 100.0% |
|  |  | % of Total | 69.5% | 25.4% | 4.5% | 0.5% | 0.1% | 100.0% |

| **Chi-Square Tests** | | | |
| --- | --- | --- | --- |
|  | Value | df | Asymptotic Significance (2-sided) |
| Pearson Chi-Square | 51.478^a^ | 4 | .000 |
| Likelihood Ratio | 48.278 | 4 | .000 |
| N of Valid Cases | 5505 |  |  |
| a. 2 cells (20.0%) have expected count less than 5. The minimum expected count is 1.45. | | | |

| **Symmetric Measures^a^** | |
| --- | --- |
|  | Value |
| N of Valid Cases | 5505 |
| a. Correlation statistics are available for numeric data only. | |

**Postprandial distress syndrome (PPDS) * Geographic**

| **Crosstab** | | | | | | |
| --- | --- | --- | --- | --- | --- | --- |
|  | | | Geographic | | | Total |
|  |  |  | Nomad life | Rural life | Urban life |  |
| Postprandial distress syndrome (PPDS) | 0 | Count | 86 | 734 | 3357 | 4177 |
|  |  | % within Postprandial distress syndrome (PPDS) | 2.1% | 17.6% | 80.4% | 100.0% |
|  |  | % within Geographic | 89.6% | 75.7% | 75.6% | 75.9% |
|  |  | % of Total | 1.6% | 13.3% | 61.0% | 75.9% |
|  | 1 | Count | 10 | 236 | 1082 | 1328 |
|  |  | % within Postprandial distress syndrome (PPDS) | 0.8% | 17.8% | 81.5% | 100.0% |
|  |  | % within Geographic | 10.4% | 24.3% | 24.4% | 24.1% |
|  |  | % of Total | 0.2% | 4.3% | 19.7% | 24.1% |
| Total | | Count | 96 | 970 | 4439 | 5505 |
|  |  | % within Postprandial distress syndrome (PPDS) | 1.7% | 17.6% | 80.6% | 100.0% |
|  |  | % within Geographic | 100.0% | 100.0% | 100.0% | 100.0% |
|  |  | % of Total | 1.7% | 17.6% | 80.6% | 100.0% |

| **Chi-Square Tests** | | | |
| --- | --- | --- | --- |
|  | Value | df | Asymptotic Significance (2-sided) |
| Pearson Chi-Square | 10.029^a^ | 2 | .007 |
| Likelihood Ratio | 11.943 | 2 | .003 |
| N of Valid Cases | 5505 |  |  |
| a. 0 cells (0.0%) have expected count less than 5. The minimum expected count is 23.16. | | | |

| **Symmetric Measures^a^** | |
| --- | --- |
|  | Value |
| N of Valid Cases | 5505 |
| a. Correlation statistics are available for numeric data only. | |

**Postprandial distress syndrome (PPDS) * Hypertension requiring medication**

| **Crosstab** | | | | | |
| --- | --- | --- | --- | --- | --- |
|  | | | Hypertension requiring medication | | Total |
|  |  |  | 0 | 1 |  |
| Postprandial distress syndrome (PPDS) | 0 | Count | 3759 | 418 | 4177 |
|  |  | % within Postprandial distress syndrome (PPDS) | 90.0% | 10.0% | 100.0% |
|  |  | % within Hypertension requiring medication | 76.7% | 68.9% | 75.9% |
|  |  | % of Total | 68.3% | 7.6% | 75.9% |
|  | 1 | Count | 1139 | 189 | 1328 |
|  |  | % within Postprandial distress syndrome (PPDS) | 85.8% | 14.2% | 100.0% |
|  |  | % within Hypertension requiring medication | 23.3% | 31.1% | 24.1% |
|  |  | % of Total | 20.7% | 3.4% | 24.1% |
| Total | | Count | 4898 | 607 | 5505 |
|  |  | % within Postprandial distress syndrome (PPDS) | 89.0% | 11.0% | 100.0% |
|  |  | % within Hypertension requiring medication | 100.0% | 100.0% | 100.0% |
|  |  | % of Total | 89.0% | 11.0% | 100.0% |

| **Chi-Square Tests** | | | | | |
| --- | --- | --- | --- | --- | --- |
|  | Value | df | Asymptotic Significance (2-sided) | Exact Sig. (2-sided) | Exact Sig. (1-sided) |
| Pearson Chi-Square | 18.332^a^ | 1 | .000 |  |  |
| Continuity Correction^b^ | 17.904 | 1 | .000 |  |  |
| Likelihood Ratio | 17.426 | 1 | .000 |  |  |
| Fisher's Exact Test |  |  |  | .000 | .000 |
| Linear-by-Linear Association | 18.329 | 1 | .000 |  |  |
| N of Valid Cases | 5505 |  |  |  |  |
| a. 0 cells (0.0%) have expected count less than 5. The minimum expected count is 146.43. | | | | | |
| b. Computed only for a 2x2 table | | | | | |

| **Symmetric Measures** | | | | | |
| --- | --- | --- | --- | --- | --- |
|  | | Value | Asymptotic Standard Error^a^ | Approximate T^b^ | Approximate Significance |
| Interval by Interval | Pearson's R | .058 | .014 | 4.288 | .000^c^ |
| Ordinal by Ordinal | Spearman Correlation | .058 | .014 | 4.288 | .000^c^ |
| N of Valid Cases | | 5505 |  |  |  |
| a. Not assuming the null hypothesis. | | | | | |
| b. Using the asymptotic standard error assuming the null hypothesis. | | | | | |
| c. Based on normal approximation. | | | | | |

**Postprandial distress syndrome (PPDS) * Diabetes Mellitus**

| **Crosstab** | | | | | |
| --- | --- | --- | --- | --- | --- |
|  | | | Diabetes Mellitus | | Total |
|  |  |  | 0 | 1 |  |
| Postprandial distress syndrome (PPDS) | 0 | Count | 3902 | 275 | 4177 |
|  |  | % within Postprandial distress syndrome (PPDS) | 93.4% | 6.6% | 100.0% |
|  |  | % within Diabetes Mellitus | 76.4% | 68.6% | 75.9% |
|  |  | % of Total | 70.9% | 5.0% | 75.9% |
|  | 1 | Count | 1202 | 126 | 1328 |
|  |  | % within Postprandial distress syndrome (PPDS) | 90.5% | 9.5% | 100.0% |
|  |  | % within Diabetes Mellitus | 23.6% | 31.4% | 24.1% |
|  |  | % of Total | 21.8% | 2.3% | 24.1% |
| Total | | Count | 5104 | 401 | 5505 |
|  |  | % within Postprandial distress syndrome (PPDS) | 92.7% | 7.3% | 100.0% |
|  |  | % within Diabetes Mellitus | 100.0% | 100.0% | 100.0% |
|  |  | % of Total | 92.7% | 7.3% | 100.0% |

| **Chi-Square Tests** | | | | | |
| --- | --- | --- | --- | --- | --- |
|  | Value | df | Asymptotic Significance (2-sided) | Exact Sig. (2-sided) | Exact Sig. (1-sided) |
| Pearson Chi-Square | 12.585^a^ | 1 | .000 |  |  |
| Continuity Correction^b^ | 12.158 | 1 | .000 |  |  |
| Likelihood Ratio | 11.906 | 1 | .001 |  |  |
| Fisher's Exact Test |  |  |  | .001 | .000 |
| Linear-by-Linear Association | 12.582 | 1 | .000 |  |  |
| N of Valid Cases | 5505 |  |  |  |  |
| a. 0 cells (0.0%) have expected count less than 5. The minimum expected count is 96.74. | | | | | |
| b. Computed only for a 2x2 table | | | | | |

| **Symmetric Measures** | | | | | |
| --- | --- | --- | --- | --- | --- |
|  | | Value | Asymptotic Standard Error^a^ | Approximate T^b^ | Approximate Significance |
| Interval by Interval | Pearson's R | .048 | .015 | 3.551 | .000^c^ |
| Ordinal by Ordinal | Spearman Correlation | .048 | .015 | 3.551 | .000^c^ |
| N of Valid Cases | | 5505 |  |  |  |
| a. Not assuming the null hypothesis. | | | | | |
| b. Using the asymptotic standard error assuming the null hypothesis. | | | | | |
| c. Based on normal approximation. | | | | | |

**Postprandial distress syndrome (PPDS) * Autoimmune diseases**

| **Crosstab** | | | | | |
| --- | --- | --- | --- | --- | --- |
|  | | | Autoimmune diseases | | Total |
|  |  |  | 0 | 1 |  |
| Postprandial distress syndrome (PPDS) | 0 | Count | 4071 | 106 | 4177 |
|  |  | % within Postprandial distress syndrome (PPDS) | 97.5% | 2.5% | 100.0% |
|  |  | % within Autoimmune diseases | 75.9% | 74.1% | 75.9% |
|  |  | % of Total | 74.0% | 1.9% | 75.9% |
|  | 1 | Count | 1291 | 37 | 1328 |
|  |  | % within Postprandial distress syndrome (PPDS) | 97.2% | 2.8% | 100.0% |
|  |  | % within Autoimmune diseases | 24.1% | 25.9% | 24.1% |
|  |  | % of Total | 23.5% | 0.7% | 24.1% |
| Total | | Count | 5362 | 143 | 5505 |
|  |  | % within Postprandial distress syndrome (PPDS) | 97.4% | 2.6% | 100.0% |
|  |  | % within Autoimmune diseases | 100.0% | 100.0% | 100.0% |
|  |  | % of Total | 97.4% | 2.6% | 100.0% |

| **Chi-Square Tests** | | | | | |
| --- | --- | --- | --- | --- | --- |
|  | Value | df | Asymptotic Significance (2-sided) | Exact Sig. (2-sided) | Exact Sig. (1-sided) |
| Pearson Chi-Square | .246^a^ | 1 | .620 |  |  |
| Continuity Correction^b^ | .157 | 1 | .692 |  |  |
| Likelihood Ratio | .242 | 1 | .623 |  |  |
| Fisher's Exact Test |  |  |  | .621 | .341 |
| Linear-by-Linear Association | .246 | 1 | .620 |  |  |
| N of Valid Cases | 5505 |  |  |  |  |
| a. 0 cells (0.0%) have expected count less than 5. The minimum expected count is 34.50. | | | | | |
| b. Computed only for a 2x2 table | | | | | |

| **Symmetric Measures** | | | | | |
| --- | --- | --- | --- | --- | --- |
|  | | Value | Asymptotic Standard Error^a^ | Approximate T^b^ | Approximate Significance |
| Interval by Interval | Pearson's R | .007 | .014 | .496 | .620^c^ |
| Ordinal by Ordinal | Spearman Correlation | .007 | .014 | .496 | .620^c^ |
| N of Valid Cases | | 5505 |  |  |  |
| a. Not assuming the null hypothesis. | | | | | |
| b. Using the asymptotic standard error assuming the null hypothesis. | | | | | |
| c. Based on normal approximation. | | | | | |

**Postprandial distress syndrome (PPDS) * Headache or migraine**

| **Crosstab** | | | | | |
| --- | --- | --- | --- | --- | --- |
|  | | | Headache or migraine | | Total |
|  |  |  | 0 | 1 |  |
| Postprandial distress syndrome (PPDS) | 0 | Count | 3808 | 369 | 4177 |
|  |  | % within Postprandial distress syndrome (PPDS) | 91.2% | 8.8% | 100.0% |
|  |  | % within Headache or migraine | 77.1% | 65.5% | 75.9% |
|  |  | % of Total | 69.2% | 6.7% | 75.9% |
|  | 1 | Count | 1134 | 194 | 1328 |
|  |  | % within Postprandial distress syndrome (PPDS) | 85.4% | 14.6% | 100.0% |
|  |  | % within Headache or migraine | 22.9% | 34.5% | 24.1% |
|  |  | % of Total | 20.6% | 3.5% | 24.1% |
| Total | | Count | 4942 | 563 | 5505 |
|  |  | % within Postprandial distress syndrome (PPDS) | 89.8% | 10.2% | 100.0% |
|  |  | % within Headache or migraine | 100.0% | 100.0% | 100.0% |
|  |  | % of Total | 89.8% | 10.2% | 100.0% |

| **Chi-Square Tests** | | | | | |
| --- | --- | --- | --- | --- | --- |
|  | Value | df | Asymptotic Significance (2-sided) | Exact Sig. (2-sided) | Exact Sig. (1-sided) |
| Pearson Chi-Square | 36.594^a^ | 1 | .000 |  |  |
| Continuity Correction^b^ | 35.968 | 1 | .000 |  |  |
| Likelihood Ratio | 34.073 | 1 | .000 |  |  |
| Fisher's Exact Test |  |  |  | .000 | .000 |
| Linear-by-Linear Association | 36.588 | 1 | .000 |  |  |
| N of Valid Cases | 5505 |  |  |  |  |
| a. 0 cells (0.0%) have expected count less than 5. The minimum expected count is 135.82. | | | | | |
| b. Computed only for a 2x2 table | | | | | |

| **Symmetric Measures** | | | | | |
| --- | --- | --- | --- | --- | --- |
|  | | Value | Asymptotic Standard Error^a^ | Approximate T^b^ | Approximate Significance |
| Interval by Interval | Pearson's R | .082 | .015 | 6.068 | .000^c^ |
| Ordinal by Ordinal | Spearman Correlation | .082 | .015 | 6.068 | .000^c^ |
| N of Valid Cases | | 5505 |  |  |  |
| a. Not assuming the null hypothesis. | | | | | |
| b. Using the asymptotic standard error assuming the null hypothesis. | | | | | |
| c. Based on normal approximation. | | | | | |

**Postprandial distress syndrome (PPDS) * Chronic immunosuppression**

| **Crosstab** | | | | | |
| --- | --- | --- | --- | --- | --- |
|  | | | Chronic immunosuppression | | Total |
|  |  |  | 0 | 1 |  |
| Postprandial distress syndrome (PPDS) | 0 | Count | 4164 | 13 | 4177 |
|  |  | % within Postprandial distress syndrome (PPDS) | 99.7% | 0.3% | 100.0% |
|  |  | % within Chronic immunosuppression | 76.0% | 56.5% | 75.9% |
|  |  | % of Total | 75.6% | 0.2% | 75.9% |
|  | 1 | Count | 1318 | 10 | 1328 |
|  |  | % within Postprandial distress syndrome (PPDS) | 99.2% | 0.8% | 100.0% |
|  |  | % within Chronic immunosuppression | 24.0% | 43.5% | 24.1% |
|  |  | % of Total | 23.9% | 0.2% | 24.1% |
| Total | | Count | 5482 | 23 | 5505 |
|  |  | % within Postprandial distress syndrome (PPDS) | 99.6% | 0.4% | 100.0% |
|  |  | % within Chronic immunosuppression | 100.0% | 100.0% | 100.0% |
|  |  | % of Total | 99.6% | 0.4% | 100.0% |

| **Chi-Square Tests** | | | | | |
| --- | --- | --- | --- | --- | --- |
|  | Value | df | Asymptotic Significance (2-sided) | Exact Sig. (2-sided) | Exact Sig. (1-sided) |
| Pearson Chi-Square | 4.727^a^ | 1 | .030 |  |  |
| Continuity Correction^b^ | 3.725 | 1 | .054 |  |  |
| Likelihood Ratio | 4.145 | 1 | .042 |  |  |
| Fisher's Exact Test |  |  |  | .047 | .032 |
| Linear-by-Linear Association | 4.726 | 1 | .030 |  |  |
| N of Valid Cases | 5505 |  |  |  |  |
| a. 0 cells (0.0%) have expected count less than 5. The minimum expected count is 5.55. | | | | | |
| b. Computed only for a 2x2 table | | | | | |

| **Symmetric Measures** | | | | | |
| --- | --- | --- | --- | --- | --- |
|  | | Value | Asymptotic Standard Error^a^ | Approximate T^b^ | Approximate Significance |
| Interval by Interval | Pearson's R | .029 | .016 | 2.175 | .030^c^ |
| Ordinal by Ordinal | Spearman Correlation | .029 | .016 | 2.175 | .030^c^ |
| N of Valid Cases | | 5505 |  |  |  |
| a. Not assuming the null hypothesis. | | | | | |
| b. Using the asymptotic standard error assuming the null hypothesis. | | | | | |
| c. Based on normal approximation. | | | | | |

**Postprandial distress syndrome (PPDS) * Anemia**

| **Crosstab** | | | | | |
| --- | --- | --- | --- | --- | --- |
|  | | | Anemia | | Total |
|  |  |  | 0 | 1 |  |
| Postprandial distress syndrome (PPDS) | 0 | Count | 3715 | 462 | 4177 |
|  |  | % within Postprandial distress syndrome (PPDS) | 88.9% | 11.1% | 100.0% |
|  |  | % within Anemia | 77.6% | 64.4% | 75.9% |
|  |  | % of Total | 67.5% | 8.4% | 75.9% |
|  | 1 | Count | 1073 | 255 | 1328 |
|  |  | % within Postprandial distress syndrome (PPDS) | 80.8% | 19.2% | 100.0% |
|  |  | % within Anemia | 22.4% | 35.6% | 24.1% |
|  |  | % of Total | 19.5% | 4.6% | 24.1% |
| Total | | Count | 4788 | 717 | 5505 |
|  |  | % within Postprandial distress syndrome (PPDS) | 87.0% | 13.0% | 100.0% |
|  |  | % within Anemia | 100.0% | 100.0% | 100.0% |
|  |  | % of Total | 87.0% | 13.0% | 100.0% |

| **Chi-Square Tests** | | | | | |
| --- | --- | --- | --- | --- | --- |
|  | Value | df | Asymptotic Significance (2-sided) | Exact Sig. (2-sided) | Exact Sig. (1-sided) |
| Pearson Chi-Square | 58.956^a^ | 1 | .000 |  |  |
| Continuity Correction^b^ | 58.239 | 1 | .000 |  |  |
| Likelihood Ratio | 54.749 | 1 | .000 |  |  |
| Fisher's Exact Test |  |  |  | .000 | .000 |
| Linear-by-Linear Association | 58.945 | 1 | .000 |  |  |
| N of Valid Cases | 5505 |  |  |  |  |
| a. 0 cells (0.0%) have expected count less than 5. The minimum expected count is 172.97. | | | | | |
| b. Computed only for a 2x2 table | | | | | |

| **Symmetric Measures** | | | | | |
| --- | --- | --- | --- | --- | --- |
|  | | Value | Asymptotic Standard Error^a^ | Approximate T^b^ | Approximate Significance |
| Interval by Interval | Pearson's R | .103 | .015 | 7.718 | .000^c^ |
| Ordinal by Ordinal | Spearman Correlation | .103 | .015 | 7.718 | .000^c^ |
| N of Valid Cases | | 5505 |  |  |  |
| a. Not assuming the null hypothesis. | | | | | |
| b. Using the asymptotic standard error assuming the null hypothesis. | | | | | |
| c. Based on normal approximation. | | | | | |

**Postprandial distress syndrome (PPDS) * Patients allergic to certain substances**

| **Crosstab** | | | | | |
| --- | --- | --- | --- | --- | --- |
|  | | | Patients allergic to certain substances | | Total |
|  |  |  | 0 | 1 |  |
| Postprandial distress syndrome (PPDS) | 0 | Count | 3770 | 407 | 4177 |
|  |  | % within Postprandial distress syndrome (PPDS) | 90.3% | 9.7% | 100.0% |
|  |  | % within Patients allergic to certain substances | 77.0% | 67.1% | 75.9% |
|  |  | % of Total | 68.5% | 7.4% | 75.9% |
|  | 1 | Count | 1128 | 200 | 1328 |
|  |  | % within Postprandial distress syndrome (PPDS) | 84.9% | 15.1% | 100.0% |
|  |  | % within Patients allergic to certain substances | 23.0% | 32.9% | 24.1% |
|  |  | % of Total | 20.5% | 3.6% | 24.1% |
| Total | | Count | 4898 | 607 | 5505 |
|  |  | % within Postprandial distress syndrome (PPDS) | 89.0% | 11.0% | 100.0% |
|  |  | % within Patients allergic to certain substances | 100.0% | 100.0% | 100.0% |
|  |  | % of Total | 89.0% | 11.0% | 100.0% |

| **Chi-Square Tests** | | | | | |
| --- | --- | --- | --- | --- | --- |
|  | Value | df | Asymptotic Significance (2-sided) | Exact Sig. (2-sided) | Exact Sig. (1-sided) |
| Pearson Chi-Square | 29.030^a^ | 1 | .000 |  |  |
| Continuity Correction^b^ | 28.491 | 1 | .000 |  |  |
| Likelihood Ratio | 27.291 | 1 | .000 |  |  |
| Fisher's Exact Test |  |  |  | .000 | .000 |
| Linear-by-Linear Association | 29.025 | 1 | .000 |  |  |
| N of Valid Cases | 5505 |  |  |  |  |
| a. 0 cells (0.0%) have expected count less than 5. The minimum expected count is 146.43. | | | | | |
| b. Computed only for a 2x2 table | | | | | |

| **Symmetric Measures** | | | | | |
| --- | --- | --- | --- | --- | --- |
|  | | Value | Asymptotic Standard Error^a^ | Approximate T^b^ | Approximate Significance |
| Interval by Interval | Pearson's R | .073 | .015 | 5.401 | .000^c^ |
| Ordinal by Ordinal | Spearman Correlation | .073 | .015 | 5.401 | .000^c^ |
| N of Valid Cases | | 5505 |  |  |  |
| a. Not assuming the null hypothesis. | | | | | |
| b. Using the asymptotic standard error assuming the null hypothesis. | | | | | |
| c. Based on normal approximation. | | | | | |

**Postprandial distress syndrome (PPDS) * Chronic obstructive pulmonary disease (COPD)**

| **Crosstab** | | | | | |
| --- | --- | --- | --- | --- | --- |
|  | | | Chronic obstructive pulmonary disease (COPD) | | Total |
|  |  |  | 0 | 1 |  |
| Postprandial distress syndrome (PPDS) | 0 | Count | 4145 | 32 | 4177 |
|  |  | % within Postprandial distress syndrome (PPDS) | 99.2% | 0.8% | 100.0% |
|  |  | % within Chronic obstructive pulmonary disease (COPD) | 76.0% | 64.0% | 75.9% |
|  |  | % of Total | 75.3% | 0.6% | 75.9% |
|  | 1 | Count | 1310 | 18 | 1328 |
|  |  | % within Postprandial distress syndrome (PPDS) | 98.6% | 1.4% | 100.0% |
|  |  | % within Chronic obstructive pulmonary disease (COPD) | 24.0% | 36.0% | 24.1% |
|  |  | % of Total | 23.8% | 0.3% | 24.1% |
| Total | | Count | 5455 | 50 | 5505 |
|  |  | % within Postprandial distress syndrome (PPDS) | 99.1% | 0.9% | 100.0% |
|  |  | % within Chronic obstructive pulmonary disease (COPD) | 100.0% | 100.0% | 100.0% |
|  |  | % of Total | 99.1% | 0.9% | 100.0% |

| **Chi-Square Tests** | | | | | |
| --- | --- | --- | --- | --- | --- |
|  | Value | df | Asymptotic Significance (2-sided) | Exact Sig. (2-sided) | Exact Sig. (1-sided) |
| Pearson Chi-Square | 3.888^a^ | 1 | .049 |  |  |
| Continuity Correction^b^ | 3.261 | 1 | .071 |  |  |
| Likelihood Ratio | 3.553 | 1 | .059 |  |  |
| Fisher's Exact Test |  |  |  | .066 | .040 |
| Linear-by-Linear Association | 3.888 | 1 | .049 |  |  |
| N of Valid Cases | 5505 |  |  |  |  |
| a. 0 cells (0.0%) have expected count less than 5. The minimum expected count is 12.06. | | | | | |
| b. Computed only for a 2x2 table | | | | | |

| **Symmetric Measures** | | | | | |
| --- | --- | --- | --- | --- | --- |
|  | | Value | Asymptotic Standard Error^a^ | Approximate T^b^ | Approximate Significance |
| Interval by Interval | Pearson's R | .027 | .015 | 1.972 | .049^c^ |
| Ordinal by Ordinal | Spearman Correlation | .027 | .015 | 1.972 | .049^c^ |
| N of Valid Cases | | 5505 |  |  |  |
| a. Not assuming the null hypothesis. | | | | | |
| b. Using the asymptotic standard error assuming the null hypothesis. | | | | | |
| c. Based on normal approximation. | | | | | |

**Postprandial distress syndrome (PPDS) * COVID-19 infection**

| **Crosstab** | | | | | |
| --- | --- | --- | --- | --- | --- |
|  | | | COVID-19 infection | | Total |
|  |  |  | 0 | 1 |  |
| Postprandial distress syndrome (PPDS) | 0 | Count | 2371 | 1806 | 4177 |
|  |  | % within Postprandial distress syndrome (PPDS) | 56.8% | 43.2% | 100.0% |
|  |  | % within COVID-19 infection | 77.2% | 74.2% | 75.9% |
|  |  | % of Total | 43.1% | 32.8% | 75.9% |
|  | 1 | Count | 699 | 629 | 1328 |
|  |  | % within Postprandial distress syndrome (PPDS) | 52.6% | 47.4% | 100.0% |
|  |  | % within COVID-19 infection | 22.8% | 25.8% | 24.1% |
|  |  | % of Total | 12.7% | 11.4% | 24.1% |
| Total | | Count | 3070 | 2435 | 5505 |
|  |  | % within Postprandial distress syndrome (PPDS) | 55.8% | 44.2% | 100.0% |
|  |  | % within COVID-19 infection | 100.0% | 100.0% | 100.0% |
|  |  | % of Total | 55.8% | 44.2% | 100.0% |

| **Chi-Square Tests** | | | | | |
| --- | --- | --- | --- | --- | --- |
|  | Value | df | Asymptotic Significance (2-sided) | Exact Sig. (2-sided) | Exact Sig. (1-sided) |
| Pearson Chi-Square | 6.960^a^ | 1 | .008 |  |  |
| Continuity Correction^b^ | 6.793 | 1 | .009 |  |  |
| Likelihood Ratio | 6.940 | 1 | .008 |  |  |
| Fisher's Exact Test |  |  |  | .008 | .005 |
| Linear-by-Linear Association | 6.959 | 1 | .008 |  |  |
| N of Valid Cases | 5505 |  |  |  |  |
| a. 0 cells (0.0%) have expected count less than 5. The minimum expected count is 587.41. | | | | | |
| b. Computed only for a 2x2 table | | | | | |

| **Symmetric Measures** | | | | | |
| --- | --- | --- | --- | --- | --- |
|  | | Value | Asymptotic Standard Error^a^ | Approximate T^b^ | Approximate Significance |
| Interval by Interval | Pearson's R | .036 | .014 | 2.639 | .008^c^ |
| Ordinal by Ordinal | Spearman Correlation | .036 | .014 | 2.639 | .008^c^ |
| N of Valid Cases | | 5505 |  |  |  |
| a. Not assuming the null hypothesis. | | | | | |
| b. Using the asymptotic standard error assuming the null hypothesis. | | | | | |
| c. Based on normal approximation. | | | | | |

**Postprandial distress syndrome (PPDS) * Open abdominal surgery/laparotomy**

| **Crosstab** | | | | | |
| --- | --- | --- | --- | --- | --- |
|  | | | Open abdominal surgery/laparotomy | | Total |
|  |  |  | 0 | 1 |  |
| Postprandial distress syndrome (PPDS) | 0 | Count | 3379 | 798 | 4177 |
|  |  | % within Postprandial distress syndrome (PPDS) | 80.9% | 19.1% | 100.0% |
|  |  | % within Open abdominal surgery/laparotomy | 77.1% | 71.1% | 75.9% |
|  |  | % of Total | 61.4% | 14.5% | 75.9% |
|  | 1 | Count | 1003 | 325 | 1328 |
|  |  | % within Postprandial distress syndrome (PPDS) | 75.5% | 24.5% | 100.0% |
|  |  | % within Open abdominal surgery/laparotomy | 22.9% | 28.9% | 24.1% |
|  |  | % of Total | 18.2% | 5.9% | 24.1% |
| Total | | Count | 4382 | 1123 | 5505 |
|  |  | % within Postprandial distress syndrome (PPDS) | 79.6% | 20.4% | 100.0% |
|  |  | % within Open abdominal surgery/laparotomy | 100.0% | 100.0% | 100.0% |
|  |  | % of Total | 79.6% | 20.4% | 100.0% |

| **Chi-Square Tests** | | | | | |
| --- | --- | --- | --- | --- | --- |
|  | Value | df | Asymptotic Significance (2-sided) | Exact Sig. (2-sided) | Exact Sig. (1-sided) |
| Pearson Chi-Square | 17.883^a^ | 1 | .000 |  |  |
| Continuity Correction^b^ | 17.554 | 1 | .000 |  |  |
| Likelihood Ratio | 17.347 | 1 | .000 |  |  |
| Fisher's Exact Test |  |  |  | .000 | .000 |
| Linear-by-Linear Association | 17.880 | 1 | .000 |  |  |
| N of Valid Cases | 5505 |  |  |  |  |
| a. 0 cells (0.0%) have expected count less than 5. The minimum expected count is 270.91. | | | | | |
| b. Computed only for a 2x2 table | | | | | |

| **Symmetric Measures** | | | | | |
| --- | --- | --- | --- | --- | --- |
|  | | Value | Asymptotic Standard Error^a^ | Approximate T^b^ | Approximate Significance |
| Interval by Interval | Pearson's R | .057 | .014 | 4.235 | .000^c^ |
| Ordinal by Ordinal | Spearman Correlation | .057 | .014 | 4.235 | .000^c^ |
| N of Valid Cases | | 5505 |  |  |  |
| a. Not assuming the null hypothesis. | | | | | |
| b. Using the asymptotic standard error assuming the null hypothesis. | | | | | |
| c. Based on normal approximation. | | | | | |

**Postprandial distress syndrome (PPDS) * (CFQ) Scale**

| **Crosstab** | | | | | | |
| --- | --- | --- | --- | --- | --- | --- |
|  | | | (CFQ) Scale | | | Total |
|  |  |  | Low fatigue | Moderate Fatigue | Severe Fatigue |  |
| Postprandial distress syndrome (PPDS) | 0 | Count | 1272 | 2590 | 315 | 4177 |
|  |  | % within Postprandial distress syndrome (PPDS) | 30.5% | 62.0% | 7.5% | 100.0% |
|  |  | % within (CFQ) Scale | 85.5% | 74.4% | 58.7% | 75.9% |
|  |  | % of Total | 23.1% | 47.0% | 5.7% | 75.9% |
|  | 1 | Count | 216 | 890 | 222 | 1328 |
|  |  | % within Postprandial distress syndrome (PPDS) | 16.3% | 67.0% | 16.7% | 100.0% |
|  |  | % within (CFQ) Scale | 14.5% | 25.6% | 41.3% | 24.1% |
|  |  | % of Total | 3.9% | 16.2% | 4.0% | 24.1% |
| Total | | Count | 1488 | 3480 | 537 | 5505 |
|  |  | % within Postprandial distress syndrome (PPDS) | 27.0% | 63.2% | 9.8% | 100.0% |
|  |  | % within (CFQ) Scale | 100.0% | 100.0% | 100.0% | 100.0% |
|  |  | % of Total | 27.0% | 63.2% | 9.8% | 100.0% |

| **Chi-Square Tests** | | | |
| --- | --- | --- | --- |
|  | Value | df | Asymptotic Significance (2-sided) |
| Pearson Chi-Square | 166.006^a^ | 2 | .000 |
| Likelihood Ratio | 164.853 | 2 | .000 |
| N of Valid Cases | 5505 |  |  |
| a. 0 cells (0.0%) have expected count less than 5. The minimum expected count is 129.54. | | | |

| **Symmetric Measures^a^** | |
| --- | --- |
|  | Value |
| N of Valid Cases | 5505 |
| a. Correlation statistics are available for numeric data only. | |

**Postprandial distress syndrome (PPDS) * Smoking**

| **Crosstab** | | | | | | | | |
| --- | --- | --- | --- | --- | --- | --- | --- | --- |
|  | | | Smoking | | | | | Total |
|  |  |  |  | A daily smoker | An occasional smoker | Ex-smoker | Non-smoker |  |
| Postprandial distress syndrome (PPDS) | 0 | Count | 1 | 649 | 457 | 218 | 2852 | 4177 |
|  |  | % within Postprandial distress syndrome (PPDS) | 0.0% | 15.5% | 10.9% | 5.2% | 68.3% | 100.0% |
|  |  | % within Smoking | 100.0% | 69.8% | 72.9% | 79.3% | 77.7% | 75.9% |
|  |  | % of Total | 0.0% | 11.8% | 8.3% | 4.0% | 51.8% | 75.9% |
|  | 1 | Count | 0 | 281 | 170 | 57 | 820 | 1328 |
|  |  | % within Postprandial distress syndrome (PPDS) | 0.0% | 21.2% | 12.8% | 4.3% | 61.7% | 100.0% |
|  |  | % within Smoking | 0.0% | 30.2% | 27.1% | 20.7% | 22.3% | 24.1% |
|  |  | % of Total | 0.0% | 5.1% | 3.1% | 1.0% | 14.9% | 24.1% |
| Total | | Count | 1 | 930 | 627 | 275 | 3672 | 5505 |
|  |  | % within Postprandial distress syndrome (PPDS) | 0.0% | 16.9% | 11.4% | 5.0% | 66.7% | 100.0% |
|  |  | % within Smoking | 100.0% | 100.0% | 100.0% | 100.0% | 100.0% | 100.0% |
|  |  | % of Total | 0.0% | 16.9% | 11.4% | 5.0% | 66.7% | 100.0% |

| **Chi-Square Tests** | | | |
| --- | --- | --- | --- |
|  | Value | df | Asymptotic Significance (2-sided) |
| Pearson Chi-Square | 30.411^a^ | 4 | .000 |
| Likelihood Ratio | 29.794 | 4 | .000 |
| N of Valid Cases | 5505 |  |  |
| a. 2 cells (20.0%) have expected count less than 5. The minimum expected count is .24. | | | |

| **Symmetric Measures^a^** | |
| --- | --- |
|  | Value |
| N of Valid Cases | 5505 |
| a. Correlation statistics are available for numeric data only. | |

**Postprandial distress syndrome (PPDS) * Alcohol**

| **Crosstab** | | | | | | | | |
| --- | --- | --- | --- | --- | --- | --- | --- | --- |
|  | | | Alcohol | | | | | Total |
|  |  |  |  | Binge Drinking | Drinking alcohol in moderation | Heavy Alcohol Use | Not drinking alcohol |  |
| Postprandial distress syndrome (PPDS) | 0 | Count | 1 | 7 | 190 | 5 | 3974 | 4177 |
|  |  | % within Postprandial distress syndrome (PPDS) | 0.0% | 0.2% | 4.5% | 0.1% | 95.1% | 100.0% |
|  |  | % within Alcohol | 100.0% | 77.8% | 78.2% | 71.4% | 75.8% | 75.9% |
|  |  | % of Total | 0.0% | 0.1% | 3.5% | 0.1% | 72.2% | 75.9% |
|  | 1 | Count | 0 | 2 | 53 | 2 | 1271 | 1328 |
|  |  | % within Postprandial distress syndrome (PPDS) | 0.0% | 0.2% | 4.0% | 0.2% | 95.7% | 100.0% |
|  |  | % within Alcohol | 0.0% | 22.2% | 21.8% | 28.6% | 24.2% | 24.1% |
|  |  | % of Total | 0.0% | 0.0% | 1.0% | 0.0% | 23.1% | 24.1% |
| Total | | Count | 1 | 9 | 243 | 7 | 5245 | 5505 |
|  |  | % within Postprandial distress syndrome (PPDS) | 0.0% | 0.2% | 4.4% | 0.1% | 95.3% | 100.0% |
|  |  | % within Alcohol | 100.0% | 100.0% | 100.0% | 100.0% | 100.0% | 100.0% |
|  |  | % of Total | 0.0% | 0.2% | 4.4% | 0.1% | 95.3% | 100.0% |

| **Chi-Square Tests** | | | |
| --- | --- | --- | --- |
|  | Value | df | Asymptotic Significance (2-sided) |
| Pearson Chi-Square | 1.156^a^ | 4 | .885 |
| Likelihood Ratio | 1.404 | 4 | .844 |
| N of Valid Cases | 5505 |  |  |
| a. 4 cells (40.0%) have expected count less than 5. The minimum expected count is .24. | | | |

| **Symmetric Measures^a^** | |
| --- | --- |
|  | Value |
| N of Valid Cases | 5505 |
| a. Correlation statistics are available for numeric data only. | |

**Postprandial distress syndrome (PPDS) * PSS Scale**

| **Crosstab** | | | | | | |
| --- | --- | --- | --- | --- | --- | --- |
|  | | | PSS Scale | | | Total |
|  |  |  | High perceived stress | Low stress | Moderate stress |  |
| Postprandial distress syndrome (PPDS) | 0 | Count | 532 | 415 | 3230 | 4177 |
|  |  | % within Postprandial distress syndrome (PPDS) | 12.7% | 9.9% | 77.3% | 100.0% |
|  |  | % within PSS Scale | 64.9% | 88.5% | 76.6% | 75.9% |
|  |  | % of Total | 9.7% | 7.5% | 58.7% | 75.9% |
|  | 1 | Count | 288 | 54 | 986 | 1328 |
|  |  | % within Postprandial distress syndrome (PPDS) | 21.7% | 4.1% | 74.2% | 100.0% |
|  |  | % within PSS Scale | 35.1% | 11.5% | 23.4% | 24.1% |
|  |  | % of Total | 5.2% | 1.0% | 17.9% | 24.1% |
| Total | | Count | 820 | 469 | 4216 | 5505 |
|  |  | % within Postprandial distress syndrome (PPDS) | 14.9% | 8.5% | 76.6% | 100.0% |
|  |  | % within PSS Scale | 100.0% | 100.0% | 100.0% | 100.0% |
|  |  | % of Total | 14.9% | 8.5% | 76.6% | 100.0% |

| **Chi-Square Tests** | | | |
| --- | --- | --- | --- |
|  | Value | df | Asymptotic Significance (2-sided) |
| Pearson Chi-Square | 96.181^a^ | 2 | .000 |
| Likelihood Ratio | 98.729 | 2 | .000 |
| N of Valid Cases | 5505 |  |  |
| a. 0 cells (0.0%) have expected count less than 5. The minimum expected count is 113.14. | | | |

| **Symmetric Measures^a^** | |
| --- | --- |
|  | Value |
| N of Valid Cases | 5505 |
| a. Correlation statistics are available for numeric data only. | |
